# A Comprehensive Analysis of Alternative Splicing in Paleopolyploid Maize

**DOI:** 10.3389/fpls.2017.00694

**Published:** 2017-05-10

**Authors:** Wenbin Mei, Sanzhen Liu, James C. Schnable, Cheng-Ting Yeh, Nathan M. Springer, Patrick S. Schnable, William B. Barbazuk

**Affiliations:** ^1^Department of Biology, University of Florida, GainesvilleFL, USA; ^2^Department of Agronomy, Iowa State University, AmesIA, USA; ^3^Department of Plant Pathology, Kansas State University, ManhattanKS, USA; ^4^Department of Agronomy and Horticulture, University of Nebraska–Lincoln, LincolnNE, USA; ^5^Department of Plant Biology, Microbial and Plant Genomics Institute, University of Minnesota, Saint PaulMN, USA; ^6^Center for Plant Genomics, Iowa State University, AmesIA, USA; ^7^Genetics Institute, University of Florida, GainesvilleFL, USA

**Keywords:** alternative splicing, maize, sorghum, seed development, abiotic stress, splicing QTL, whole genome duplication

## Abstract

Identifying and characterizing alternative splicing (AS) enables our understanding of the biological role of transcript isoform diversity. This study describes the use of publicly available RNA-Seq data to identify and characterize the global diversity of AS isoforms in maize using the inbred lines B73 and Mo17, and a related species, sorghum. Identification and characterization of AS within maize tissues revealed that genes expressed in seed exhibit the largest differential AS relative to other tissues examined. Additionally, differences in AS between the two genotypes B73 and Mo17 are greatest within genes expressed in seed. We demonstrate that changes in the level of alternatively spliced transcripts (intron retention and exon skipping) do not solely reflect differences in total transcript abundance, and we present evidence that intron retention may act to fine-tune gene expression across seed development stages. Furthermore, we have identified temperature sensitive AS in maize and demonstrate that drought-induced changes in AS involve distinct sets of genes in reproductive and vegetative tissues. Examining our identified AS isoforms within B73 × Mo17 recombinant inbred lines (RILs) identified splicing QTL (sQTL). The 43.3% of *cis-*sQTL regulated junctions are actually identified as alternatively spliced junctions in our analysis, while 10 Mb windows on each side of 48.2% of *trans*-sQTLs overlap with splicing related genes. Using sorghum as an out-group enabled direct examination of loss or conservation of AS between homeologous genes representing the two subgenomes of maize. We identify several instances where AS isoforms that are conserved between one maize homeolog and its sorghum ortholog are absent from the second maize homeolog, suggesting that these AS isoforms may have been lost after the maize whole genome duplication event. This comprehensive analysis provides new insights into the complexity of AS in maize.

## Introduction

Alternative splicing (AS) is a mechanism for generating multiple isoforms from a single pre-mRNA by the regulated selection of splice sites during mRNA processing. Recent estimates indicate that about 95% of multi-exon genes in the human genome undergo AS (Pan et al., 2008), in contrast to 61% in *Arabidopsis* (Marquez et al., 2012). While exon skipping is the dominant AS event in animals, intron retention events prevail in plants (Barbazuk et al., 2008). AS is regulated by both *cis-*regulatory elements and *trans-*elements such as hnRNP and SR proteins (Reddy et al., 2012). AS not only contributes to increased protein diversity (Graveley, 2001; Kazan, 2003), but also may regulate protein levels by influencing relative isoform ratios (Wang et al., 2008). Additionally, the study of *UPF* (up-frameshift) mutants has suggested that purposeful production of unproductive messages by AS in combination with nonsense-mediated decay is a strategy used for post-transcriptional regulation of gene expression by plants (Kalyna et al., 2012; Drechsel et al., 2013).

Maize has been the focus of extensive genetics and genomics analysis as well as being an important crop. The maize genome has been sequenced and assembled (Schnable et al., 2009) and much recent effort has been given toward gene expression studies, variant detection, epigenetics, and whole genome association studies (Barbazuk and Mei, 2014). The present work focuses on the use of publicly available RNA-Seq data to characterize AS in B73 inbred maize. Herein, we describe a strategy for assembling and characterizing AS isoforms from short-read RNA-Seq data and apply this to define and characterize a genome-wide collection of B73 transcripts and AS events. Using this collection of AS events, we investigate differential AS of pre-mRNA transcripts between 14 tissues in B73 maize.

Maize exhibits enormous genetic diversity, including extensive copy number variation and presence–absence variation (PAV) among inbred lines (Springer et al., 2009; Swanson-Wagner et al., 2010; Hirsch et al., 2014). Utilizing publicly available Mo17 RNA-Seq we investigate transcriptome diversity and characterize B73 and Mo17 genotype specific differential AS within five maize tissue types. Evidence suggests that there are extensive AS changes during development and in response to environmental stresses in plants (Staiger and Brown, 2013). Thatcher et al. (2016) discovered AS in maize during drought stress in ear, tassel, and leaf tissues, and in addition identified differential splicing during seed development. We complement this analysis by identifying genes that undergo differential AS under temperature and drought stress between tissues in B73, under mild and severe drought at different durations in B73, and under temperature stress across B73 and Mo17. Our specific focus on characterizing the biological relevance of the extensive intron-retention events common to AS in plants reveals that intron retention is regulated across seed development independent of total transcript abundance, and may serve a role in fine-tuning gene expression. Genetic variation has been shown to influence splicing regulation in plants (Zhang et al., 2011; Kesari et al., 2012; Thatcher et al., 2014). A previous study in maize using the intermated B73×Mo17 (IBM) Syn10 DH population mapped 235 genes, whose splicing were *cis-* and *trans-*regulated (Thatcher et al., 2014). We apply a novel splice junction-based approach to define and examine *cis-* and *trans-*QTLs using RNA-Seq data from a second collection of IBM recombinant inbred lines (RILs; Li et al., 2013) to further characterize the genetic basis of AS.

Maize experienced a whole genome duplication (WGD) about 5–12 million years ago, subsequent to its divergence from the last common ancestor shared with sorghum (Swigonova et al., 2004). Two subgenomes within maize have been reconstructed using a comparative genomics approach (Schnable et al., 2011). A recent study showed subgenome1 has been under purifying selection driven by tissue subfunctionalization (Pophaly and Tellier, 2015). In addition, 13% of maize homeolog gene pairs are proposed to have undergone regulatory neofunctionalization in leaf (Hughes et al., 2014). So far no attempt has been made to investigate AS in homeolog pairs within maize subgenomes. We present a comparative genomics analysis of AS between maize homeologs and their orthologous genes in sorghum that identified conserved AS events, and revealed instances of AS absence in one versus the other maize homeolog that likely represent AS loss after the maize WGD event.

## Materials and Methods

### Sequence Collection and Processing

We downloaded 27,455 Maize inbred line B73 full-length cDNAs from the maize cDNA project^1^ and 765,748 ESTs from NCBI. We used SeqClean^2^ to clean full-length cDNAs and ESTs based on universal vector database. The 109,217 B73 ESTs were discarded due to low quality or short read length (<100 bp). In addition, we downloaded over 8.1 billion publicly available B73 Illumina RNA-Seq reads from 18 different tissues (Supplemental Data 1) and B73 10 PacBio SMRT cells long reads from seedlings (SRA053579). The 288,849 raw Mo17 ESTs were retrieved from NCBI and 164,956 were kept after quality trimming. In addition, we collected 1.9 billion Mo17 RNA-Seq reads from 12 different tissues (Supplemental Data 1). To determine *cis-* or *trans-*sQTL, we used 3.47 billion public RNA-Seq data from a population of 105 B73×Mo17 RILs (Li et al., 2013). The 106,603 sorghum sanger ESTs were collected from NCBI and 797 million Illumina RNA-Seq reads from 40 sorghum samples (Dugas et al., 2011; Olson et al., 2014). The 39,001 sorghum full-length cDNA data were downloaded from DDBJ (DRX027767) (Shimada et al., 2015). Adapters were removed from raw RNA-Seq reads with Cutadapt v1.1 (Martin, 2011) followed by low quality filtering with Trimmomatic v0.32 (Bolger et al., 2014) requiring a minimum length of 25 bp and otherwise default settings. Trimmed reads were aligned to the B73 RefGen_v2 in GSNAP version 20141216 (Wu and Nacu, 2010) with parameters “-s -B 5 –suboptimal-levels = 0 -m 0.05 -n 1 -Q -N 1 -w –novelend-splicedist -J 33 -j 0 –nofails –sam-multiple-primaries –pairmax-rna” and using known maize splice sites detailed in RefGen_v2. Duplicate reads were removed with Picard v1.115 MarkDuplicates^3^ and only unique alignments for single end reads and concordant unique alignments for paired end reads were kept for further analysis.

### Transcriptome Assembly and AS Isoform Detection

Three transcript assembly platforms were used to maximize isoform detection: (i) Cufflinks 2.2.1 (Trapnell et al., 2012) with parameters “–GTF-guide –max-intron-length 8000 -b -F 0.05 –no-faux-reads”; (ii) genome guided Trinity 2.0.4 (Grabherr et al., 2011) with parameters “–genome_guided_max_intron 8000”; (iii) and StringTie 1.0.0 (Pertea et al., 2015) with parameters “-G -f 0.05 -j 2”. The collection of Cufflinks isoforms with ≥0.1 FPKM, StringTie, and Trinity defined isoforms, together with filtered FLcDNA and ESTs were merged and aligned back to the maize B73 RefGen_v2 with GMA (Wu and Watanabe, 2005) and clustered with PASA 2.0 (Haas et al., 2003) to remove redundancy. We further filtered splice isoforms by requiring: (1) splice junctions within the transcripts were confirmed with splicing entropy score above 2 calculated using Spanki v0.5.0 with default parameters (Sturgill et al., 2013); (2) retained introns had a minimum median read coverage of 10 over a minimum of 90% of the intron length and a minimal intron retention percentage of 10% (Marquez et al., 2012); (3) each isoform had a minimum FPKM of 1 and represented at least 5% of the isoform fraction in at least one tissue examined. Supplemental Figure 1 illustrated the details of this pipeline.

### Classifying AS Events and Evaluating Coding Potential

Five types of AS events (intron retention, alternative acceptor site, alternative donor site, exon skipping, and alternate terminal exon) were considered for this study. Candidate coding regions of alternatively spliced isoforms were identified using Transdecoder^4^, and protein domains were identified with HMMER3 (Eddy, 1998). We defined isoforms that may lead to Nonsense Mediated Decay (Lewis et al., 2003) as those with premature termination codons (PTCs) that share a start site with the longest canonical transcript from the annotation and have PTCs farther than 50 bp upstream of last exon–exon junction. We paid special attention to maize transcription factor genes, splicing related genes and ‘classic’ loci identified by mutation and genetically characterized by the maize community. Maize transcription factor families were obtained from GrassTFDB (Yilmaz et al., 2009). Maize splicing related genes were obtained from Splicing Related Gene Database (SRGD) in PlantGDB (Duvick et al., 2008) and the coordinates were converted from maize B73 RefGen v1 to v2. We are focusing on three categories of splicing related genes based on the classification in SRGD: small nuclear ribonucleoprotein (snRNP), splicing factors and splicing regulation genes. Maize ‘classical’ loci were obtained from CoGe (Schnable and Freeling, 2011).

### *In Silico* Validation of AS Using PacBio Reads

The B73 Illumina reads from the same study together with PacBio long reads (Martin et al., 2014) were quality trimmed and *in silico* normalized with Trinity, and then used to error correct PacBio long reads using three iterations of LSC 1.alpha (Au et al., 2012). Corrected PacBio reads longer than 500 bp and aligned to B73 RefGen_v2 along ≥90% of their length with ≥95% identity in PASA were compared to transcripts assembled from RNA-Seq short reads with Cuffcompare v2.2.1. In house scripts were used to assess differences in splice junctions.

### Identification of AS across B73 Maize Tissues

We utilized two strategies to examine differences in AS. Differential AS between tissues in B73, within a given tissue but between genotypes, or between genotypes or tissues under stress were detected by assaying for differentially spliced isoforms between tissues. This analysis considered all five AS types characterized above. Isoforms from a given gene are differentially spliced if their relative ratios of abundance differ between the samples compared. Differences in the relative ratios of isoforms between tissues, for example, indicate tissue specific differences in RNA processing decisions. Note that differential AS does not necessarily reflect changes in overall expression at the locus. Differential splicing was reported with the Cuffdiff utility from the Cufflinks v2.2.1 package.

A second methodology was applied to examine changes in exon skipping and intron retention isoforms to profile changes in these two AS classes across different tissues in B73. For these cases the intron retention ratio (IRR) of retained introns and the percentage splice in (PSI) of skipped exons were determined. Similar to Florea et al. (2013) isoforms of each AS event were classified into an on or off mode. In the skipped exon event, the canonical exon-containing splice isoform is “on” and the exon skipped isoform is “off.” In an intron retention event the intron retention isoform is “on” and the canonical intron splice isoform is “off.” The IRR or PSI is = ΣFPKM_on_/(ΣFPKM_on__+_ ΣFPKM_off_) where FPKM_on_ and FPKM_off_ represent the “on” and “off” gene expression levels for the appropriate AS isoforms calculated in Cufflinks 2.2.1 defining the exon skipping (or intron retention) events. If there are more than two isoforms that support the same intron retention or exon skipping event, we sum up the expression levels for all “on” isoforms and all “off” isoforms. We computed the IRR for intron retention isoforms, or the PSI for exon skipping isoforms, using isoforms that exhibited a minimum FPKM of 1 across all tissues. Those intron retention or exon skipped isoforms with IRR or PSI values between 0.05 and 0.95 across all samples, respectively, were subjected to Ward’s hierarchical clustering (Ward, 1963).

The smallest data set from the 18 tissues examined (Supplemental Data 2) was from pericarp (20M unique alignments after removing duplicates). To rule out bias from different read depths among datasets, 20M unique alignments were randomly selected from each of the remaining tissues and the IRR and PSI values determined. A Pearson correlation analysis between IRR and PSI values from the random subsampling alignments and IRR and PSI values calculated from all data for each tissue was performed. The minimum Pearson correlation values for IRR and PSI between subsampling and all data in each tissue were 0.97 and 0.96, respectively (Supplemental Figures 2, 3).

We selected 9 out of 18 tissues [ear, embryo, endosperm, leaf, root, shoot apical meristem (SAM), seed, seeding and tassel] based on their large sequence collections (*n* ≥ 121M de-duplicated uniquely mapped reads) and multiple independent samples (*n* ≥ 14) and calculated pairwise differential splicing differences among these tissues using the Cuffdiff utility from Cufflinks 2.2.1 (Supplemental Data 2). The differentially spliced isoforms were further filtered to require at least one isoform in each condition above an FPKM of 5, and at least one isoform must be more abundant in one of the two conditions compared.

### AS Profiling during Seed Development and in Response to Stress

Alternative splicing was profiled during seed development from day 0 to day 38 using RNA-Seq data from a previous study (Chen et al., 2014). We utilized IRR values as previously described and required ΔIRR values to be Δ(38 day–0 day) > 0.15, with the additional requirement that intron retention isoforms have FPKM ≥ 1 and IRR between 0.05 and 0.95 from day 0 to day 38. Hierarchical clustering of the ΔIRR values was based on Ward’s method using the Python seaborn.clustermap function available within the seaborn package^5^. We also compared the gene expression values between day 0 and day 38 from those loci with ΔIRR > 0.15 using a Student’s *t*-test. Similar to the AS comparison performed between tissues (above) differential splicing was identified and compared using RNA-Seq data (Makarevitch et al., 2015) that was acquired from maize seedlings under controlled temperature, cold stress (incubated at 5°C for 16 h) and heat stress (50°C for 4 h). Similarly, RNA-Seq data (Kakumanu et al., 2012) was used to assess differential splicing between ovary (reproductive tissue) and leaf (vegetative tissue) under well-watered and drought conditions. Finally, RNA-Seq data (Opitz et al., 2014) was used to identify genes undergoing differential splicing between seedling primary roots under mild and severe water deficit relative to well-watered controls at two time periods 6 and 24 h. All the stress related and seed development RNA-Seq data in B73 is described in Supplemental Data 1.

### Identifying AS Differences between Inbred Lines B73 and Mo17

Alternative splicing isoforms in Mo17 were constructed using publicly available Mo17 RNA-Seq data from 12 tissues (Supplemental Data 2) using the same methods described to detect AS in B73 with the additional use of a SNP tolerant GSNAP alignment step (option -use-snps) For this, previously described B73/Mo17 sequence variants (Paschold et al., 2012) were taken into consideration to correct for mapping bias expected from aligning Mo17 RNA-Seq reads to the B73 reference. The identified Mo17 AS isoforms were merged with the previously identified B73 isoforms to assess genotype specific AS detection. Differential AS between B73 and Mo17 was examined in five tissues that had deep RNA-Seq collections from B73 and Mo17 including leaf, shoot apical meristem, root, seed, and seedling tissues (Supplemental Data 2). Additionally, the splicing differences between B73 and Mo17 during cold and heat response were compared using AS isoforms constructed with RNA-Seq data (Makarevitch et al., 2015).

### Splicing-QTL Analysis in the Inter-mated B73 × Mo17 Recombinant Inbred Lines (IBM RILs) Population

RNA-seq reads from B73 × Mo17 RILs and their B73 and Mo17 parents from (Li et al., 2013) were aligned to the B73 reference genome (AGPv2) with both GSNAP and Tophat (Trapnell et al., 2009). We identified 221,764 splice sites within 19,955 genes from the B73 RefGen_v2 filtered gene set by both GSNAP and Tophat. Of these, 126,189 splice sites from 18,819 genes were supported by a minimum of 20 spanning reads and were considered ‘high-confidence.’ The 91,063 splice sites were common to both the *de novo* detected ‘high-confidence’ splice site sets described above. A subset of the overlapping splicing sites (*N* = 73,956) each of which was supported by five reads per line on average and known annotated introns were considered during subsequent sQTL analysis.

To identify *cis-*splicing QTL (*cis-*sQTL) and *trans-*splicing QTL (*trans-*sQTL) for each intron the IBM RILs individuals were divided into two groups based on their genotype scoring of *cis-*markers and *trans-*markers, respectively. Genetic markers are genomic segments each of which harbors multiple single nucleotide polymorphisms (SNPs). Each segment in each RIL exhibits a consistent genotype, either B73 or Mo17, across all SNPs on the segment (Liu et al., 2012). For each splicing site, the genotype of a *cis*-marker is the genotype of the segment in which the splicing site is located. *Trans-*markers are located on different chromosomes relative to the associated splice sites. The 4,892 markers were used in *trans-*sQTL analysis. To minimize the potential bias due to the alignment preference of reads from the B73 allele to the B73 reference genome. For each splicing junction, the read count of the junction was corrected by the count of other reads that were not on the junction but on the same gene. The null hypothesis is that there is no difference in the number of reads spanning a given splice site between B73 and Mo17, which is determined by comparing the relative expression of a given splice site to the total expression of the gene containing the splice site, between two RIL groups defined by genotyping scores suggestive of either a *cis*-marker or a *trans*-marker. Differences in splice site read counts normalized by the underlying gene expression were tested using a binomial model with the overdispersion control. We used the *glm* function implemented in R to detect differences in splicing frequency between two RIL groups at each splice site. Conceptually, the splicing frequency is the proportion of splicing (intron-spanning) reads of a given splicing site among the total number of reads from the corresponding gene. A false discovery rate (FDR) correction was applied to account for multiple tests (Benjamini and Hochberg, 1995) associated with *cis*-sQTL. Given the expensive computation required for the *trans-*sQTL study and the independence of markers within chromosomes, neither a standard permutation test, nor a FDR method was used during the *trans-*sQTL analysis. Instead, a modified Bonferroni multiple test correction (Li et al., 2013) was used to obtain the *p*-value cutoff for extracting significant *trans-*sQTLs. Briefly, each chromosome was divided into 100 equal sized regions and all markers within a given region were merged and treated as a single marker to reduce total number of tests. The final *p*-value cutoff, 6.15*e* - 09, was determined through dividing 0.05 by the total number of tests. The tests showing *p*-values lower than this final cutoff were considered as significant *trans-*sQTLs. To identify enrichment of SNP polymorphisms near splice sites, 9 bp 5′ (CAGGURAGU) and 3 bp 3′ (AGG) segments that include each splice site were assessed for SNPs previously defined between B73 and Mo17 (Paschold et al., 2014). In addition, 1 and 10 Mb sequence segments flanking *trans-*sQTL were examined for the presence of known splice related gene loci using BEDTools (Quinlan and Hall, 2010).

### Comparison of AS between Homeologs in the Maize Subgenomes and Their Orthologs in Sorghum

Orthologous gene sets between maize subgenome1, subgenome2, and sorghum were identified using previously described methodology (Schnable et al., 2011). Alternative splice isoforms within maize subgenome1 and subgenome2 were identified within the global set of B73 isoforms, and sorghum AS isoforms were detected using the pipeline described above for AS detection in B73. Conserved AS events were recognized by identifying the junctions flanking the AS event within each AS isoform (e.g., the 5′ and 3′ flanking junctions of an alternatively spliced exon within an exon-skipping isoform), and extracting up to 300 bp of anchor nucleotide sequence on both sides of the splice junctions. We termed these as splice anchor sequence tags (SAST). Collections of SASTs from maize subgenome1, 2 and sorghum were constructed for each of the five splice events examined, The SAST from the same AS events in maize subgenome1, 2 and sorghum were tBLASTx aligned using WU-BLAST (Gish, W. 1996–2003)^6^ and only alignments with an *E*-value of 1*e* - 5 or lower were considered. Alignments between SASTs that were derived from members of the same triplet gene group defined by maize subgenome1 and subgenome2 homeologs and their sorghum ortholog defined conserved AS events. Alignments between SASTs less than 30 bp in two of the members were not considered. Additionally, the *E* ≤ 1*e* - 5 criteria was relaxed if well aligned SASTs were less than 100 bp in length. Conserved AS events within maize subgenome1, subgenome2, and sorghum were classified into four different categories; (1) Conserved between both maize subgenome1 and subgenome2 homeologs, and their sorghum ortholog; (2) Conserved between the maize subgenome1 homeolog and sorghum, but absent from the maize subgenome2 homeolog; (3) Conserved between the maize subgenome2 homeolog and sorghum, but absent from the maize subgenome1 homeolog; and, (4) Conserved between both maize subgenome1 and subgenome2 homeologs but absent from the sorghum ortholog).

### GO Term Enrichment

GO term enrichment analysis was performed using the agriGO analysis toolkit (Du et al., 2010). We used the Fisher exact test to detect enriched GO categories, with multiple testing correction using the Hochberg FDR method with a minimal adjusted *p*-value of 0.05.

## Results

### Nearly 60% of Expressed Multi-exon Genes in B73 Exhibit Evidence of AS

**FIGURE 1:**
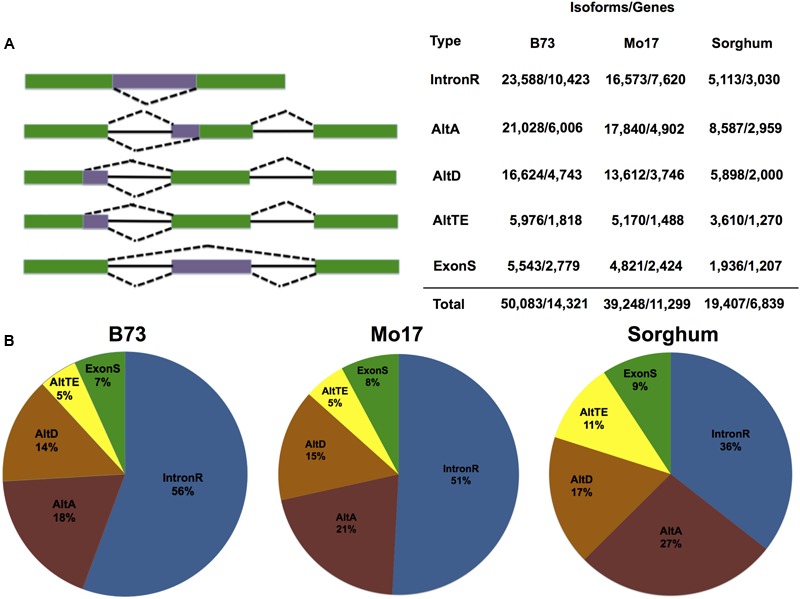
**Genome-wide AS event distributions and patterns. (A)** Graphic of event types followed by the number of AS isoforms and the number of genes that demonstrate this type of splicing event in maize inbred lines B73 and Mo17, and sorghum. **(B)** Proportions of alternative splicing events (alternative acceptor, AltA; alternative donor, AltD; Exon Skipping, ExonS; Intron Retention, IntronR; alternate terminal exon, AltTE) found in maize inbred lines B73 and Mo17, and sorghum.

The 8.1 billion public RNA-Seq reads from 18 B73 tissues were used to identify AS in B73. A B73 transcriptome was assembled using each of three reference guided short-read transcript assembly strategies with the approximately 3.4 billion reads that remained after quality filtering and removal of non-unique aligned reads (see Materials and Methods). The assembled transcripts constructed by each assembler were pooled along with 522,672 Sanger sequenced ESTs (>200 bp) and 27,412 full-length cDNAs available from GenBank, and clustered with PASA (Haas et al., 2003). PASA clustering resulted in 157,767 transcripts defining 31,027 multi-transcript loci, and these were further filtered based on splice junction coverage, intron coverage and isoform expression and AS event type. Only those isoforms representing intron retention, alternative acceptor, alternative donor, exon skipping or alternate terminal exon AS events that overlap with B73 RefGen_v2 gene loci were considered. In total, we identified 50,083 isoforms within 14,321 gene loci (**Figure 1A**), which represents 46.6 and 59.7% of expressed multi-exon genes in the working and filtered gene sets, respectively – note that one gene could have multiple AS events. In total, we identified 53,692 AS events in B73 (Supplemental Table 1).

### *In Silico* Validation of Short-Read AS Transcript Assemblies with PacBio Long Reads

To assess the accuracy of isoform and splice junctions constructed from short read data, short read assemblies were evaluated against long reads from 10 PacBio SMRT cells made available by Martin et al. (2014). The PacBio long reads were error corrected by LSC (Au et al., 2012) using the Illumina short read data from the same study (Martin et al., 2014). The 6,468 PacBio reads longer than 500 bp were aligned to B73 RefGen_v2 along ≥90% of their length with ≥95% identity and clustered into 2,480 multi-exon transcript isoforms by PASA. The 8,862 splice junctions were defined by these 2,480 isoforms, of which 8,850 (99.8%) exist within our RNA-Seq splice junction database and 8,766 (98.9%) were represented within our short-read transcript assemblies. The 95.2% of the 2,480 PacBio defined isoforms were identical to (1,053 isoforms), or contained within (1,307 isoform) transcripts assembled by short reads. The remaining 120 are potentially novel.

### Functional Characterization of AS Isoforms

The 13,529 out of 14,321 genes that produce AS transcripts are annotated as protein coding genes in the RefGen_v2 working gene set; 281 of these are annotated as pseudo-genes; 511 are annotated as transposable elements. Consistent with other reports (Thatcher et al., 2014; Chamala et al., 2015) intron retention was the most commonly observed AS event (**Figure 1B**) and accounts for 23,588 (47.1%) of predicted AS isoforms (Supplemental Table 1). This is followed by alternative acceptor, alternative donor, alternate terminal exon and exon skipping. PASA prediction update the UTR regions of the final set of 50,083 assembled AS transcript isoforms revealed that 41.6% of the AS events occurred in the coding region, and 27.6 and 30.8% occur within 5′and 3′ UTR regions, respectively (Supplemental Figure 4A). The 45.4% of intron retention events impact the coding region, which is the highest among five AS types (Supplemental Figure 4A). In general, 1/3 of the AS events preserve reading frame, which is similar to results expected for stochastic splicing (Satyawan et al., 2016); however, approximately 40% of predicted exon skipping and alternative acceptor events maintain the original reading frame suggesting that at least some AS events are not random splicing errors (Supplemental Figure 4B).

The impact of the B73 maize AS events on protein domains was assessed by examining changes to the predicted coding regions of AS isoforms *in silico*. The proportion of AS transcripts from each event type predicted to alter protein products is greatest for alternate terminal exon events (33.2%), while approximately 20% for the others (Supplemental Figure 4C). Predicting the translation start site within assembled AS transcripts can be error-prone, particularly if the transcript is not 5′ complete (Brown et al., 2015). Considering those isoforms that have identical translational start sites to the longest transcripts previously reported within the reference annotation identifies that 33,634 (67%) isoforms contain a start site identical to the original annotation and 15,455 (31%) of these also contain PTCs (Supplemental Figure 4D; Nagy and Maquat, 1998). The 10,236 (66%) of the PTC containing isoforms are the result of intron retention while 1,619 (10%) PTC isoforms are the result of exon skipping.

Transcription factors are important regulators of development and stress response. Members of several transcription factor families including bHLH, bZIP, NAC, MYB, Homeobox, WRKY, and MYB-related exhibit AS (Supplemental Figure 5), and the functional importance of some AS isoforms of TF genes have been characterized (Szécsi et al., 2006). In addition, many plant genes that function to regulate AS, such as splice factors and components of the spliceosome, also undergo extensive AS (Braunschweig et al., 2013). For example, our analysis determined that 19/21 maize SR protein genes undergo AS. Overall, our analysis suggests that similar proportions of maize snRNP and splicing factor genes undergo AS, while a greater proportion of splicing regulator genes undergo AS relative to snRNP and splicing factor genes (Supplemental Table 2). Among the 446 maize loci that represent ‘classical maize genes’ (Schnable and Freeling, 2011) 184 produce AS transcripts. The details of AS events associated with these genes are described in Supplemental Data 3. Some AS among these ‘classical’ loci had been previously discovered and characterized further emphasizing the important roles that AS isoforms play in plant growth and development [e.g., AS of the *waxy* loci (GRMZM2G024993) changing the amylose content in the rice cultivars (Varagona et al., 1992) and AS at *r1* (GRMZM5G822829)] – a *bHLH* transcription factor involved in anthocyanin biosynthesis (Procissi et al., 2002).

### Splicing Differences between Tissues and Development Stages

**FIGURE 2:**
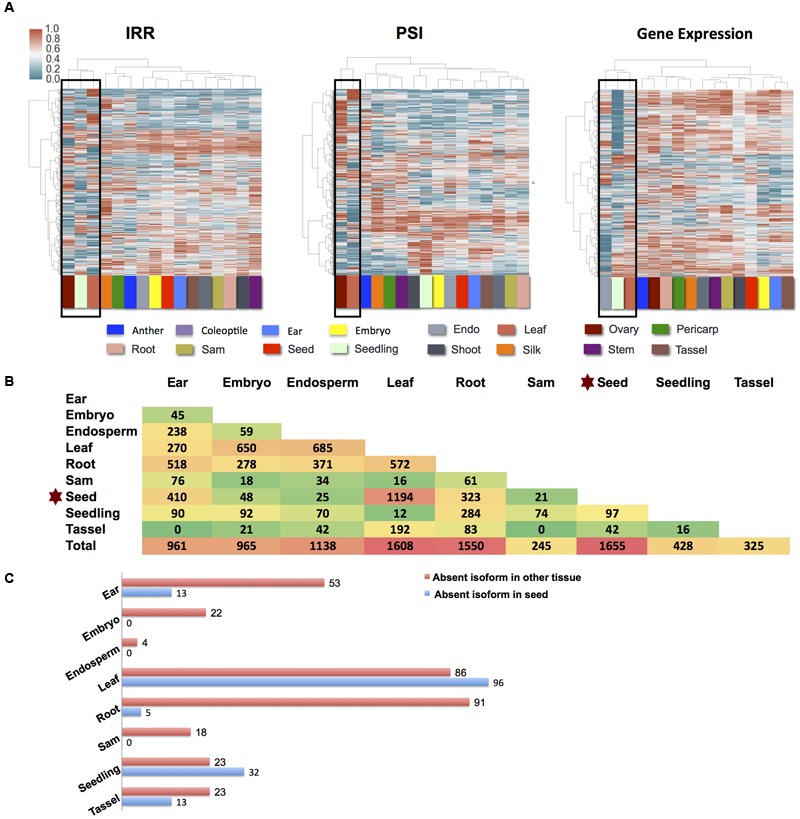
**AS difference among different tissues. (A)** Characterization IRR, PSI, and gene expression across 16 different B73 tissues. Intron retention and exon skipping isoforms presented required an FPKM ≥ 1 IRR or PSI values between 0.05 and 0.95 across all tissues. All genes presented in the expression analysis panel were required to have FPKM ≥ 1. Hierarchical clustering was performed using the Ward’s method after a log transform. Outlying clusters are boxed. **(B)** Number of differential splicing genes for pairwise CuffDiff comparison among nine tissues in B73. Results were filtered to ensure that at least one isoform for each gene was present at FPKM ≥ 5 in each condition and that at least one isoform for each gene is higher expressed in each condition. Considering the variability and multiple datasets, FPKM 5 was chosen to be stringent to filter noise in the differential splicing results. **(C)** Based on the differential splicing results between seed and other tissues (B and Supplemental Data 4) potential present and absent (PAV) isoforms can be detected. Those isoforms with expression levels >2 FPKM in one tissue and <0.1 in the other were considered to be PAV.

Starch endosperm and aleurone have a noticeably different number of AS genes and AS frequencies among different AS types relative to the other 16 tissues for which data was available, and these were removed from further analyses (Supplemental Figures 6A,B). The remaining 16 B73 tissues were used to examine tissue specific differential AS (see Materials and Methods), and correlation analysis suggested that read number differences between these data sets would not bias expression results (see Materials and Methods). An unsupervised clustering analysis was performed on the expression of exon skipping events (PSI value), intron retention events (IRR value) and overall gene expression and used to infer similarities and differences in exon skipping, intron retention and overall expression between tissues (**Figure 2A**). Similar to the results from Maize Gene Expression Atlas (Stelpflug et al., 2015), leaf tissue expression was part of an outlying cluster in all three clustering conditions. Seedling expression data is represented within the outlying group in both IRR and gene expression clustering but not in the PSI clustering. Ovary expression data represents an outlier in terms of IRR and PSI, but does not cluster within an outlying group based on in gene expression suggesting that reproductive tissue (ovary) has a distinct tissue specific splicing pattern. Differential splicing examined in a pair-wise fashion between nine tissues (ear, embryo, endosperm, leaf, root, SAM, seed, seedling, and tassel) suggests that genes expressed in the seed undergo the highest levels of differential splicing relative to the remaining tissues examined (**Figures 2B,C**). The list of genes exhibiting differential splicing in pairwise comparisons among the nine tissues is described in Supplemental Data 4. It is noteworthy that the number of multi-exon genes that produce alternatively spliced transcripts is highest in reproductive tissues: ovary and anther (Supplemental Figure 6A), yet these tissues do not cluster together. This suggests that despite a shared proclivity for AS, maize male and female reproductive tissues probably have distinct splicing profiles suggestive of distinct mRNA processing programs. Here, we show that the patterns of AS splicing in maize are often distinct from gene expression patterns, which is also observed in the human Genotype-Tissue Expression (GTEx) project (Ardlie et al., 2015).

**FIGURE 3:**
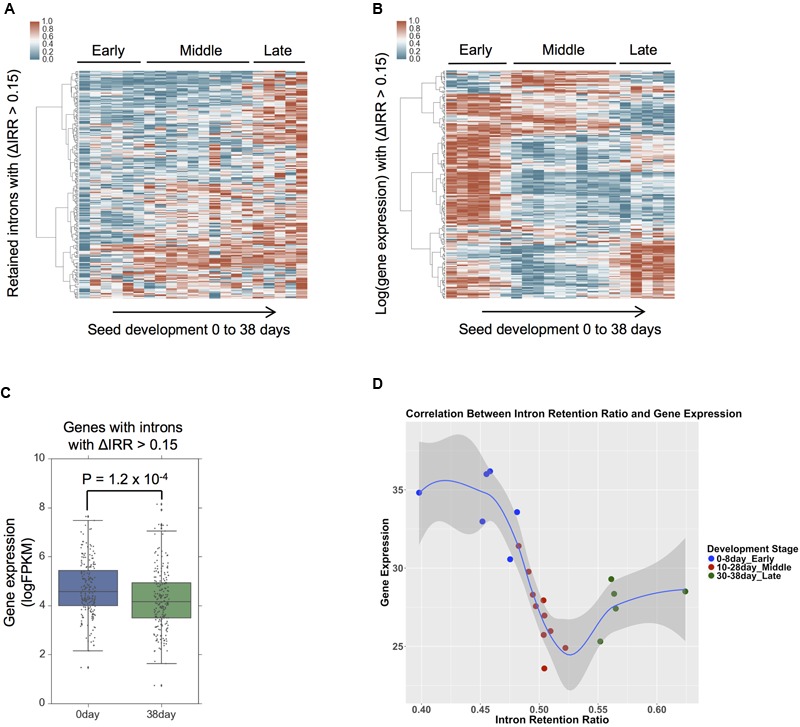
**Intron retention dynamics during seed development. (A)** Intron retention isoforms require a minimum FPKM of 1 and intron retention ratio (IRR) values between 0.05 and 0.95 from 0 to 38 days across all seed development stages. Hierarchical clustering the IRR values Δ(day 38–day 0) > 0.15 using the Ward’s method. **(B)** Identification of gene expression values for those intron retention events in **(A)**, hierarchical clustering was based on log transformed gene expression values. **(C)** A comparison of expression levels at seed development 0 and 38 day from genes that produce intron retention events with IRR values Δ(38 day–0 day) > 0.15, *p*-value was calculated by Student’s *t*-test. **(D)** Gene expression and IRR trends across different stages of seed development. Each point represents a sample. The average IRR from genes that exhibit IR (ΔIRR > 0.15) is plotted against their average gene expression across development.

To examine the relationship between AS and gene expression during seed development, 242 intron retention events with IRR values that differ by a minimum of 0.15 between day 38 and day 0 were selected, and their IRR changes examined during seed development from day 0 to day 38 (**Figure 3A**). The IRR profiles of these 242 intron retention events indicates that IRR values gradually increase from early to late development giving rise to three distinct expression clusters consistent with the definition of the seed development stages (Chen et al., 2014). Hierarchical clustering of the expression of the 209 genes associated with these 242 intron retention events indicates that their expression shows distinct patterns during development (**Figure 3B**) with significant differences among gene expression values (*P* = 1.2 × 10^-4^, Student’s *t*-test) when comparing only day 0 to day 38 (**Figure 3C**). GO term enrichment analyses suggest these genes are over-represented for kinase and phosphotransferase activity (Supplemental Figure 7). The negative correlation between gene expression and intron retention values is observed between early and middle seed development stages, and also between early and late development stages (**Figure 3D**). However, this negative correlation is not evident between middle and late development stages (**Figure 3D**). This result suggests that, similar to the role of intron retention during neuron differentiation, terminal erythropoiesis and CD4+ T cell activation (Braunschweig et al., 2014; Ni et al., 2016; Pimentel et al., 2016), intron retention in maize may fine-tune gene function during seed development by further reducing the expression of low abundance transcripts that are no longer required, particularly during the early and middle seed development stages (0–28 days) that are characterized by cellularization and differentiation (Sabelli and Larkins, 2009).

### AS Response to Temperature and Drought

**FIGURE 4:**
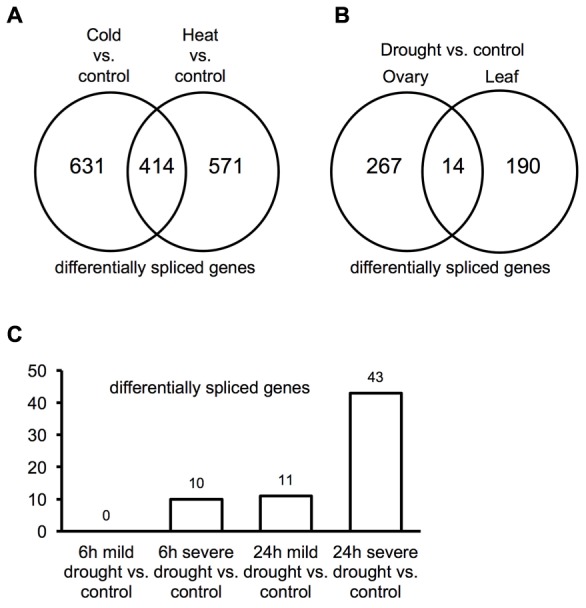
**AS associated with stress response. (A)** Differentially spliced genes within seedlings induced in response to cold or heat treatment. **(B)** Differentially spliced genes within ovary vs. leaf induced in response to drought treatment. **(C)** Differences in the numbers of differentially spliced genes within root tissue subjected to mild vs. severe drought for short (6 h) vs. long (24 h) durations.

Plants alter splicing patterns in response to temperature stress (Filichkin et al., 2010; James et al., 2012; Chang et al., 2014; Capovilla et al., 2015). Differential AS in association with temperature stresses was examined in maize using RNA-Seq data from seedlings (Makarevitch et al., 2015). Analysis of this data identified 1,045 maize genes that exhibit differential splicing between cold treated maize tissues and controls. Additionally, 985 maize genes exhibit differential splicing between heat-stressed plant tissues and controls with 414 of these common to both heat and cold responses (**Figure 4A**). GO enrichment analysis applied to genes exhibiting differential splicing common to both heat and cold stress identified an enrichment in genes involved in regulatory processes (Supplemental Figure 8). Of the 21 SR protein genes in maize, five and four genes were differentially spliced in response to cold and heat, respectively (Supplemental Data 5); two of these SR protein genes, zm-SC30 (GRMZM2G016296) and zm-SCL30 (GRMZM2G065066), are predicted to produce splice isoforms in response to both cold and heat stresses that differ from control temperature samples. Among 40 hnRNP or hnRNP related genes, four and one genes show differential splicing in response to cold and heat, respectively (Supplemental Data 5). Two hnRNP A/B family genes (GRMZM2G139643 and GRMZM2G167356) and two hnRNP genes with homologs in animals (GRMZM2G177001 = CUG-BP1-2 and GRMZM2G103430 = hnRNP-R1) exhibit differential spicing in response to cold treatment, while only one hnRNP gene (GRMZM2G467907) exhibits differential splicing during heat stress. These results suggest that SR and hnRNP genes respond differently to temperature stresses.

Drought is a complex abiotic process that has both short and long duration effects on plants. Tissues are affected at separate rates and exhibit different responses to drought (Thatcher et al., 2016). Re-analyzing data from Kakumanu et al. (2012) identified 281 and 204 genes that exhibit differential splicing during drought stress within ovary and leaf, respectively. Only 14 of these genes are common to both tissues suggesting that reproductive and vegetative tissues respond differently to drought stress and this is accomplished, in part, through differential AS (**Figure 4B**). It takes time for plants to respond to drought, thus it seems reasonable to expect that both the length and severity of the drought period may elicit differing effects that would influence how plants respond to drought. RNA-Seq data from roots (Opitz et al., 2014) subjected to drought for two durations (i.e., 6 and 24 h) and two intensities (i.e., mild and severe) were examined (**Figure 4C**). No differential splicing was detected at 6 h under mild drought, but 10 genes exhibited differential splicing when subjected to severe drought for 6 h. When the duration of drought was increased to 24 h, 11 genes exhibited differential gene splicing under mild drought, while 43 genes were differentially spliced under severe drought conditions. Seven genes that exhibit differential splicing were shared by mild and severe drought. These results suggest that the regulation of splicing differs between early and late drought response and these differences may relate to differences in the physiological response associated with these different stress conditions. There is one gene that undergoes differential AS that is shared between 6 and 24 h severe drought response but not detected in mild drought response: *cyc3* (GRMZM2G073671). *cyc3* is a maize cyclin gene that plays a role during the G2/M transition of the cell cycle (Renaudin et al., 1994). Type B cyclin genes are maximally expressed during the G2/M transition and M phase (Kakumanu et al., 2012). Drought has been shown to disrupt the cell cycle, and inhibits cell proliferation by down-regulating cell cycle genes in leaf and ovary tissue (Kakumanu et al., 2012; Avramova et al., 2015). Interestingly, during mild or severe, 6 or 24 h drought the expression level of the *cyc3* isoform without the retained intron (GRMZM2G073671_asmbl_272716) is greatly reduced (almost zero) (Supplemental Table 3), while expression of GRMZM2G073671 decreased as well during drought stress. Intron retention may act to switch from a functional *cyc3* to a non-functional *cyc3* transcript under drought stress, which may assist in halting the cell cycle and slowing growth. Genes exhibiting differential splicing in response to stresses described above are described further in Supplemental Data 6.

### Differential Splicing between Maize Inbreds

**FIGURE 5:**
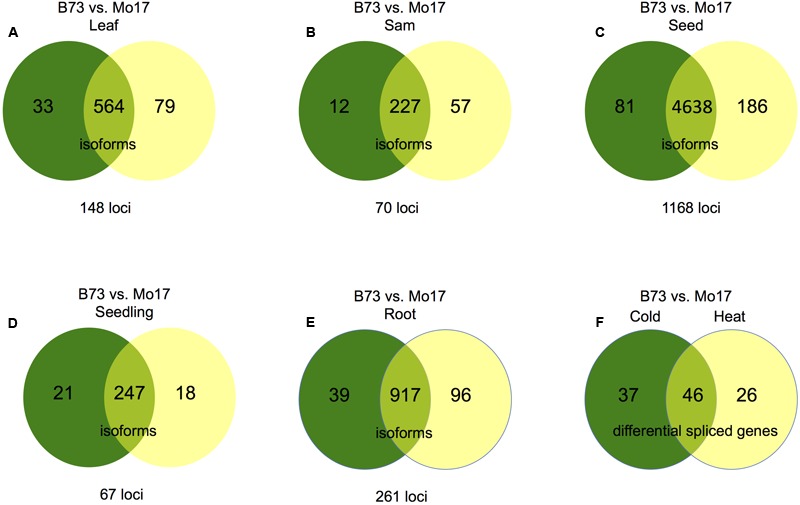
**Genotype specific AS between B73 and Mo17.** For figures **(A–E)** numbers in the left portion of the Venn diagrams (green) represent isoforms absent in B73 but present in Mo17; numbers in the right portion of the Venn diagrams (yellow) represent isoforms absent in Mo17 but present in B73. Numbers in the overlap regions represent isoforms follow other patterns, but also in differentially spliced genes. **(A)** Differential AS between B73 and Mo17 in leaf tissue. **(B)** Differential AS between B73 and Mo17 in SAM tissue. **(C)** Differential AS between B73 and Mo17 in seed tissue. **(D)** Differential AS between B73 and Mo17 in seedling tissue. **(E)** Differential AS between B73 and Mo17 in root tissue. **(F)** Genes that undergo differential AS between B73 and Mo17 in response to cold and heat stress. The number of genes that are differentially spliced between Mo17 and B73 under cold stress that are not responsive to heat stress are presented on the left. The number of genes that are differentially spliced between Mo17 and B73 under heat stress that are not responsive to cold stress are presented on the right, The number of genes that are differentially spliced between Mo17 and B73 under both heat and cold stress are presented at the interface.

Alternative splicing was identified in maize Mo17 using the pipeline described previously (Materials and Methods). Fewer alternatively spliced isoforms were detected in Mo17 than in B73, potentially due to less data in Mo17 (**Figure 1A**), although the distribution of different types of AS events is similar (**Figure 1B**) and intron retention is also the predominant AS type (Supplemental Table 1). Cuffdiff analysis between Mo17 and B73 for each tissue, or treatment was performed (see Materials and Methods, **Figures 5A–E** and Supplemental Data 7) and seeds exhibited the greatest difference in AS; 1,168 genes expressed in seed exhibited differential AS between B73 and Mo17 (**Figure 5C**). Additionally, B73 was compared to Mo17 under both heat and cold stress to identify genes that are differentially spliced in response to either stress. There were 83 genes that exhibit differential AS between B73 and Mo17 under cold stress and 72 genes that exhibit differential AS between B73 and Mo17 under heat stress. Among these two differential spliced gene lists, 46 genes were in common (**Figure 5F**) suggesting that the stress response mechanisms for both heat and cold partially overlap between genotypes while also demonstrating genotype specific differences in AS in response to cold and heat at these loci.

### Genetic Control of AS: Identification of Splicing QTL

**FIGURE 6:**
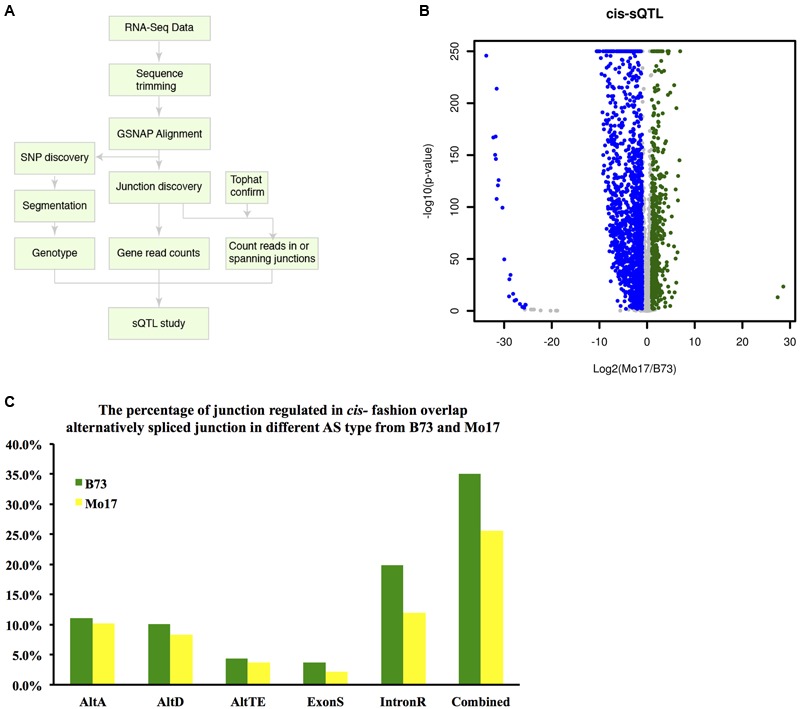
***Cis*-sQTL analysis. (A)** Flow chart describing the splicing QTL analysis. **(B)** *Cis-*sQTL results. Blue indicates higher expression in B73 (more intron junction read coverage), while green indicates higher expression in Mo17 (more intron junction read coverage). **(C)** Overlap between *cis-*sQTL junction and the alternatively spliced junction in each AS events in genotype B73 and Mo17.

A high confidence collection of splice sites was identified as illustrated in **Figure 6A**; each is supported by at least 20 intron-spanning reads. In total, we modeled 73,956 splice junctions in the sQTL analysis that were supported by both GSNAP and Tophat splice alignment (See Materials and Methods). To identify sQTL, a binomial model was used to test the null hypothesis that no difference exists in the splicing odds at each of these splice sites among the IBM RILs sorted into B73-type and Mo17-type RILs at each genetic marker. To identify *cis-*sQTL, 73,957 splice junctions supported on average by at least five reads per RIL were examined. At an FDR of 5%, 20,708 (28%) of the tested splice junctions exhibited a significant difference in splicing odds between B73-type and Mo17-type RILs. Using a more stringent criterion that required at least a two-fold change in splicing odds, splicing of 565 and 1,437 introns were up-regulated and down-regulated in Mo17 relative to B73, respectively (**Figure 6B**). To validate the *cis-*sQTL, 2,002 significant splice junctions were overlapped with the splice junctions identified earlier in the analysis. The 701 (35.0%) and 512 (25.6%) *cis-*sQTL splice junctions, respectively, overlap with B73 and Mo17 alternatively spliced junctions identified with our AS identification methods (**Figure 6C**). The 43.3% of *cis*-sQTL splice junctions overlapped with merged alternatively spliced junctions between B73 and Mo17, which indicates our *cis*-sQTL analysis indeed captured a large proportion of alternative spliced junctions. In addition, SNP polymorphisms between B73 and Mo17 around each splice junction were examined. Among the 2,002 *cis-*sQTL junctions that exhibited at least two-fold change in splicing odds, 179 (8.9%) contain polymorphisms within a 12 bp region including 9 bp region downstream of the 5′ splice site (CAGGURAGU) and 3 bp region upstream of the 3′ splice site (AGG). In comparison, only 2.6% (1,940/73,957) of all splice junctions used to examine *cis-*sQTL harbor polymorphic SNPs. Polymorphisms around splice junctions between B73 and Mo17 are significantly enriched at *cis-*sQTL splice junctions (*P* < 0.0001, Chi-square test with Yates correction).

**FIGURE 7:**
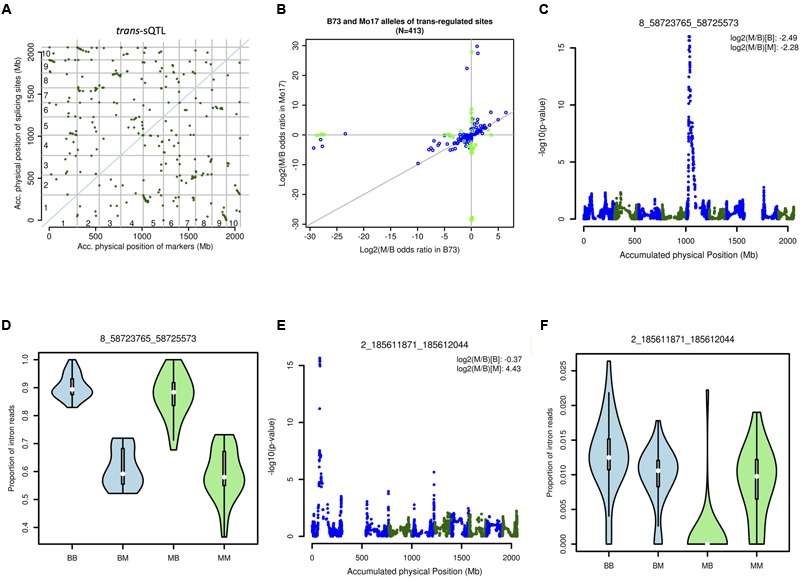
***Trans*-sQTL analysis. (A)** Distribution of *trans-*sQTL on the maize chromosomes. **(B)** Interaction between the marker and the junction. Blue: significant *trans-*effect but not significant *cis- trans-* interaction. Green: significant *trans-*effect and significant *cis- trans-* interaction. **(C)** QTL peak associated with the splice junction chr8_58723765_58725573. **(D)** Expression patterns for junction chr8_58723765_58725573 associated with four different allelic contributions (two alleles for the marker and two alleles for the junctions, the first letter represents the allele for marker and the second letter represents the allele for junction): BB, BM, MB, and MM. **(E)** QTL peak associated with the splice junction chr2_185611871_185612044. **(F)** Expression patterns for splice junction chr2_185611871_185612044 associated with four different allelic contributions two alleles for the marker and two alleles for the junctions, the first letter represents the allele for marker and the second letter represents the allele for junction): BB, BM, MB, and MM.

The genetic associations between each marker on all chromosomes other than the chromosome on which a given splice junction is located were examined to identify *trans-*sQTL. Altogether, more than 322 million statistical tests were performed. Interaction between *cis-* and *trans*-markers were included in the statistical model. In total, 7,672 pairs of splice junction by statistically significant *trans*-sQTL were detected. Based on final *p*-value 6.15*e* - 09 accounting for multiple tests, 413 *trans-*regulated splice junctions and their *trans*-sQTLs were identified (**Figure 7A**). In additional, 43 *trans*-regulated splice junctions showed significant interactions between genotype of the markers and splice junctions (**Figure 7B**). Some *trans-*sQTLs are regulated similarly between B73-type and Mo17-type marker genotypes, such as the *trans-*regulation of an intron in the zinc finger family protein (GRMZM2G056524; **Figures 7C,D**). Some *trans-*sQTLs are regulated only by the Mo17-type marker genotype but not the B73-type, such as the splice junction at position chr2_185611871_185612044 in GRMZM2G077718 (**Figures 7E,F**). It has been hypothesized that this type of interaction could contribute to hybrid vigor (Schnable and Springer, 2013). We examined the overlap of the *trans-*sQTLs with known splicing-related genes (see Materials and Methods). The 86 (25.9%) and 160 (48.2%) *trans*-sQTLs are located within 1 and 10 Mb distance to a splicing related gene, respectively. The overlap frequencies were similar to previous discovery based on isoform ratio analysis (Thatcher et al., 2014) and also suggested that much more hidden “splicing code” remains to be discovered.

### Examining AS in the Subgenomes of Maize and Sorghum to Identify Lost and Novel Isoforms

We identified 15,744 AS events in sorghum with intron retention the most predominant AS type (Supplemental Table 1). These AS events are distributed within 19,407 isoforms from 6,839 genes (**Figure 1A**). A comparative genomic analysis defined 6,489 gene triplets composed of maize subgenome1 and subgenome2 homeologous genes and their sorghum ortholog (see Materials and Methods). The 4,121 triplets remain after filtering out those containing tandem duplicates and requiring at least two of three triplet genes to be multi-exon and expressed. Within these 4,121 triplets, 2,093 (50.8%) subgenome1 gene members, 2,108 (51.2%) subgenome2 gene members and 1,290 (31.3%) sorghum gene members exhibit AS. The 91 (7.1%) sorghum genes produce alternative splice isoforms that are also conserved within the corresponding subgenome1 and subgenome2 homeologous gene pairs, while 326 (25.3%) of sorghum genes produce alternatively spliced isoforms within either the corresponding region of subgenome1 or subgenome2. Combined, these data identify 375 (29.1%) out of 1,290 sorghum AS genes conserved with maize. There is no detectable bias in AS between subgenome1 and subgenome2, nor in the number of conserved alternative splice isoforms from subgenome1 or subgenome2 with sorghum, despite reported expression biases associated with the maize subgenomes (Schnable et al., 2011). There are 235 genes (11.2%) from the 2,093 alternatively spliced subgenome1 genes that have a conserved alternatively spliced isoform with their sorghum ortholog, and 246 genes (11.8%) from the 2,108 alternatively spliced subgenome2 genes that have a conserved alternatively spliced isoform with their sorghum ortholog (**Table 1**). This result agrees with results in wheat; no bias in percentage of AS was detected between subgenomes (Akhunov et al., 2013). Given that sorghum and modern maize share a common ancestor approximately 12 MYA (Swigonova et al., 2004), the instances of conserved isoforms between the subgenome1 homeolog and the sorghum ortholog where the isoform is absent from the subgenome2 homeolog; or, conserved isoforms between subgenome2 homeolog and the sorghum ortholog where the isoform is absent from the subgenome1 homeolog, likely represent instances where this isoform existed within a lineage ancestral to maize and sorghum but was lost in one or the other maize homeologs subsequent to the maize WGD. Likewise, some of the events present in either (but not both), maize subgenomes and absent from sorghum may represent events that were acquired after the WGD event in *Zea mays*. Finally, there are 259 genes with alternative splice isoforms that are conserved between the subgenome1 and subgenome2 homeologs, but absent from the sorghum orthologs. While we cannot rule out the possibility that some events were missed as a result of incomplete sampling in sorghum, it is possible that some of the alternatively spliced isoforms present in either or both subgenomes of maize but absent in sorghum represent maize specific events that evolved after the divergence of maize and sorghum lineages. However, it is also possible that some of these events were shared within a common ancestor and subsequently lost in sorghum. The most likely origin of these events would require assessment of conserved splicing in additional out-group lineages.

**Table 1:** Conserved AS between maize subgenomes and sorghum.

		All conserved			Maize1 Sorghum		Maize2 Sorghum		Maize1 Maize2		Total conserved	
		Maize1	Maize2	Sorghum	Maize1	Sorghum	Maize2	Sorghum	Maize1	Maize2	Maize^∗^	Sorghum^∗^
IntronR	Clusters	40	40	40	84	84	68	68	265	265	192	192
	Events	47	48	51	96	97	79	79	303	299	270	227
	Genes	36	36	36	74	74	65	65	213	213	203	165
AltA	Clusters	39	39	39	57	57	65	65	32	32	161	161
	Events	49	52	50	65	68	78	76	42	42	244	194
	Genes	38	38	38	55	55	64	64	31	31	193	150
AltD	Clusters	19	19	19	35	35	38	38	36	36	92	92
	Events	23	21	19	41	50	45	40	44	38	130	109
	Genes	19	19	19	35	35	38	38	36	36	111	92
ExonS	Clusters	9	9	9	16	16	17	17	11	11	42	42
	Events	9	10	9	16	17	17	17	11	11	52	43
	Genes	8	8	8	16	16	16	16	11	11	48	39
AltTE	Clusters	0	0	0	11	11	6	6	6	6	17	17
	Events	0	0	0	14	13	10	10	9	6	24	23
	Genes	0	0	0	11	11	6	6	6	6	17	17
Total	Clusters	107	107	107	203	203	194	194	350	350	504	504
	Events	128	131	129	232	245	229	222	409	396	720	596
	Genes	94	94	94	172	172	173	173	273	273	505	395

*Values are reported for clusters, events, and genes. ^∗^Total conserved in maize represents clusters, events, or genes within maize1 and maize2 from all conserved, maize1 from maize1 sorghum conserved and maize2 from maize2 sorghum conserved.*

## Discussion

This study describes a rigorous computational method to identify and characterize AS transcripts from short-read RNA-Seq data. Our methods demonstrate the ability to rapidly assemble and characterize AS isoforms from short RNA-Seq that can be applied to any species with available RNA-Seq and a draft genome assembly. Although short-read transcript assembly is still challenging (Pertea et al., 2015), PacBio Iso-Seq is relatively low throughput, costly, and requires deep sampling to recover rare isoforms and perform expression analysis (Sharon et al., 2013) and may be better suited for generating a reference transcriptome catalog that can support quantitative analyses with short-read data sets (Wang et al., 2016). Junctions detected within the maize Iso-Seq data produced by Martin et al. (2014) are largely represented within our assembled transcripts. Recently, a second published collection of B73 Iso-Seq data (Wang et al., 2016) has been described and is substantially increased in transcripts and isoforms relative to the Martin et al. (2014) collection, but this also required substantial investment, is limited to a small number of tissues and provides a qualitative analysis of transcript/isoform diversity. The short-read RNA-Seq isoform assembly methodology described herein provides a useful method to assess transcript isoform diversity across tissues, genotypes and treatments using any public RNA-Seq data available for organisms with reference genome sequences, although one must remain aware that short-read assembly can be error prone (Steijger et al., 2013).

Alternative splicing is regulated by the “splicing code” (Wang and Burge, 2008; Reddy et al., 2012) consisting of *cis-*regulatory elements such as exonic splicing enhancers and silencers (Fu and Ares, 2014) and intronic splicing enhancers and silencers (Yeo et al., 2007), and *trans-*regulatory elements (Busch and Hertel, 2012). In this study, we identified similarities and differences between gene expression patterns and splicing between tissues (**Figure 2A**). Seed has a greater abundance of genes that undergo differential splicing than the other 15 tissues assessed. This observation underscores earlier reports characterizing splicing in developing seed that suggests splicing plays an active role (Sugliani et al., 2010; Fouquet et al., 2011), and by our observation that the abundance of intron-retention splice isoforms changes as seeds develop. Similar to Thatcher et al. (2014) who suggested that single tissue specific isoforms are rare in maize, most instances of differential splicing we observed result in changes in the relative abundance of isoforms that are expressed within both tissues being compared, and strict tissue specific isoform expression, detected as presence/absence of particular isoforms, is relatively rare (**Figure 2C** for seed vs. all other tissues; Supplemental Table 4).

We identified a subset of genes whose expression during seed early and middle development stages is negatively correlated with IRR, which is a measure of the abundance of intron retention isoform(s) relative to constitutively spliced intron isoform(s) from a locus (**Figures 3A–D**). To our knowledge, this is the first study in plants demonstrating this exact relationship between gene expression and intron retention. Shen et al. (2014) classified soybean genes into high AS genes or low AS genes based on the number of AS isoforms and compared their expression levels, while Thatcher et al. (2016) identified and examined differential splicing and differential gene expression in seed, endosperm and embryo across development. However, neither of these studies reported a direct relationship between intron retention and gene expression. Braunschweig et al. (2014) reported a negative correlation between gene expression and intron retention during neuron differentiation in embryonic stem cells, and suggested that intron retention may fine-tune gene function during differentiation by further reducing the expression of low abundance transcripts that may be no longer necessary in the tissues they are detected in. In these cases, expression is not entirely shut-off, but splicing is regulated to increase production of non-functional isoforms. This analysis suggests that an analogous process may be occurring during maize seed development. During cell differentiation in seed development, intron retention may fine-tune protein levels by reducing the levels of functional transcripts from loci whose products are irrelevant to the specific seed tissue or developmental time point they are expressed in, without the need to shut down transcription entirely. A similar mechanism is utilized by the TFIIIA locus in plants (Fu et al., 2009; Barbazuk, 2010). GO enrichment analysis on the set of genes that increase intron retention abundance during seed development suggests enriched signaling and cell communication (Supplemental Figure 7). Shunting message processing toward the production of non-functional transcript isoforms that are subject to nonsense mediated decay has the potential to regulate protein levels post-transcriptionally (Lewis et al., 2003; Merkin et al., 2012). Thus, it is possible that intron retention is being used during seed maturation or cell differential stages to shut down or reduce signaling pathways that are no longer necessary.

Our examination of AS isoforms and differential splicing in heat or cold stressed maize identified 414 loci that undergo differential splicing during both heat and cold response. This number reflects a substantial portion of all loci observed to undergo differential splicing in response to cold (39.6%) or heat (42.0%) treatment. These genes probably reflect loci that undergo AS in a temperature dependent manner. GO term analysis of these 414 genes uncovers an enrichment of genes regulating cellular and metabolic processes and protein transport (Supplemental Figure 8). Recently it has been suggested that AS allows plants to rapidly modulate the abundance of functional transcripts in response to changing environmental conditions (Capovilla et al., 2015) including ambient temperature change (Streitner et al., 2013). Our characterization of cold/heat related splicing is the first report that identifies core sets of genes in maize that may be acting in this fashion. Five and four SR genes were found to undergo differential splicing in response to cold or heat stress, respectively; two SR genes, zm-SC30 (GRMZM2G016296) and zm-SCL30 (GRMZM2G065066), are differentially splicing during both treatments. An additional intron is generated in the 3′ UTR region of zm-SC30. Interestingly, that 3′ UTR intron splice isoform introduces a PTC leading to the nonsense mediated decay, and this intron retention event in the 3′ UTR is evolutionary conserved across different plant species (Rauch et al., 2014). We detected the same intron retention event in the 3′ UTR (Supplemental Figure 9) and further demonstrate that expression level of two isoforms (GRMZM2G016296_asmbl_188362 and GRMZM2G016296_asmbl_188364, which spliced that 3′ UTR intron), are decreased during stress (Supplemental Table 4). These two isoforms (GRMZM2G016296_asmbl_188362 and GRMZM2G016296_asmbl_188364) would lead to nonsense mediate decay based on *in silico* prediction. The expression level of these two isoforms are highest in the control sample, and the ratio for the sum of expression levels of these two isoforms relative to total gene expression decrease under cold, heat, salt or UV stress (Supplemental Table 4). Other isoforms which do not splice that 3′ UTR intron have increased expression under stress. For example, the levels of isoforms GRMZM2G016296_asmbl_188361 and GRMZM2G016296_asmbl_188366 increase under response to all stresses examined except UV (Supplemental Table 4). Upon UV stress, the level of GRMZM2G016296_asmbl_188365, which also does not have that 3′ UTR intron spliced out (Supplemental Table 4), increased substantially compared to the control condition. This observation is similar to the *Arabidopsis* gene *ATSRP30*, which also decreases the expression of an unproductive PTC+ isoform and increases expression of a productive functional isoform in response to stress (Filichkin et al., 2010). This kind of switch between productive and unproductive isoforms might be common for SR genes. These results further demonstrate that differential splicing occurs in response to cold and heat stress. Taking into consideration studies that demonstrated AS isoforms participating in regulating circadian clock (James et al., 2012) and flowering time (Posé et al., 2013) in response to temperature change, breeding programs that are focused on temperature stress tolerant varieties may need to consider better characterizing temperature related splicing and establishing its association with improved temperature stress, particularly for breeding cold tolerant maize varieties.

Our examination of differential splicing during drought treatment identified splicing differences in reproductive ovary and vegetative leaf tissue in response to drought, and also revealed that short periods of drought (6 h) and extended drought (24 h) result in different splicing responses in seedling roots. This implies that there is more than one pathway activated by drought in different tissues, or that prolonged drought results in additional changes over short drought periods. Remarkably, *cyc3* shows an increase in intron retention isoform production during short and prolonged drought, suggesting the possibility that drought induced changes in *cyc3* may be influencing slow-down or cessation of the cell cycle. Perhaps increasing expression of intron retention isoforms, most of which are presumably non-functional, from genes involved in cell cycle and basic cell metabolism are a general hallmark of stress response acting to slow cell growth. Additional examination of the roles AS may play in cell cycle genes in response to stress is required.

Genetic polymorphisms that disrupt splicing regulation can lead to human disease (Xiong et al., 2015) or impact plant development as seen for rough endosperm 3 (*rgh3*) in maize (Fouquet et al., 2011; Gault et al., 2017). Examining AS isoform expression within several tissues (i.e., root, SAM, seedling, and leaf) between the genotypes B73 and Mo17 revealed that genes undergoing differential splicing were most abundant in seed. This is consistent with results from pair-wise tissue comparisons within B73 and intron retention expression profiling across seed development, which demonstrate the presence of extensive AS in seed tissue. In addition to characterizing splice isoforms in B73 and Mo17, we further explored *cis-* and *trans-*regulation of AS among IBM RILs. A previous study based on seedling leaf tissue from IBM RILs grown hydroponically (Thatcher et al., 2014), focused on genetic mapping those isoforms that exhibited differential splicing between B73 and Mo17. Here, we developed a junction-based approach using seedling shoot apices to identify 2,012 splice junctions with large *cis-*effects and 413 splice junctions that are under *trans*-regulation. The 8.9% *cis-*sQTL junctions have SNPs within a 12 bp region including 9 bp region downstream of the 5′ splice site (CAGGURAGU) and 3 bp region upstream of the 3′ splice site (AGG) that are significantly enriched. However, polymorphisms near the splicing junctions explain only a small proportion of *cis*-sQTLs. There are many other aspects of the splicing code such as exonic splicing enhancers/silencers and intronic splicing enhancers/silencers that are found within the introns or exons they regulate, but at some distance from the splice sites (Reddy et al., 2012).

The 11.2 and 11.8% genes that undergo AS in maize subgenome1 and subgenome2, respectively, produce an AS isoform that is conserved in an orthologous sorghum gene. Likewise, 29.1% of sorghum genes produce an AS isoform that is conserved in at least one of the maize subgenome 1 or 2 homeologs. This level of conservation is likely an underestimate and may increase with deeper RNA-Seq sampling of sorghum. At any rate, our findings based on available data suggest that the majority of sorghum genes with evidence of AS (70.9%) do not have conserved AS events with at least one of the two maize homeologs that make up an orthologous triplet. Had we considered the cases defined by a sorghum – maize orthologous gene pair that result from the presence of only one maize homeolog (presumably the result of loss of one maize homeolog after the maize WGD), the proportion of genes sharing conserved AS between sorghum and maize may be larger. We also observed instances that are most easily explained by the loss of a splicing event from one of the two maize homeologs subsequent to the allotetraploidy event – perhaps through a fractionation process that involves isoform loss rather than whole locus loss. Similar losses of AS events have been described within the eudicot species soybean and *Phaseolus* (Chamala et al., 2015).

The analyses presented here made use of existing large scale RNA-Seq datasets that were developed for other purposes and placed into the public domain. Thus, we expect that these will have certain limitations in sampling relative to data sets that were specifically acquired for our analysis purposes and are not expected to be completely comprehensive in their ability to discover and characterize AS isoforms. Nevertheless, this analysis presents a robust and re-usable discovery pipeline and provides the maize community with immediate access to the characterized maize AS collection via FigShare^7^ to use in their own research. Our results provide valuable insight into the differences in splicing among tissues, genotypes, seed development, and during or following stress. The future availability of additional RNA-Seq datasets will increase our understanding of the role of AS in plant development, stress response, gene regulation and adaptation.

## Author Contributions

WM, WB, SL, NS, and PS designed the work. WM, WB, SL, JS, and C-TY analyzed the data. WM and WB wrote the manuscript with input from SL, JS, NS, and PS.

## Conflict of Interest Statement

The authors declare that the research was conducted in the absence of any commercial or financial relationships that could be construed as a potential conflict of interest.

## Footnotes

1 http://www.maizecdna.org/

2 https://sourceforge.net/projects/seqclean/

3 http://broadinstitute.github.io/picard/

4 https://transdecoder.github.io/

5 http://seaborn.pydata.org/generated/seaborn.clustermap.html

6 http://blast.wustl.edu

7 https://doi.org/10.6084/m9.figshare.4205079.v3

## References

[B1] Akhunov E. D. Sehgal S. Liang H. Wang S. Akhunova A. R. Kaur G. (2013). Comparative analysis of syntenic genes in grass genomes reveals accelerated rates of gene structure and coding sequence evolution in polyploid wheat. *Plant Physiol.* 161 252–265. 10.1104/pp.112.205161 23124323 PMC3532256

[B2] Ardlie K. G. Deluca D. S. Segrè A. V. Sullivan T. J. Young T. R. Gelfand E. T. (2015). The genotype-tissue expression (GTEx) pilot analysis: multitissue gene regulation in humans. *Science* 348 648–660. 10.1126/science.1262110 25954001 PMC4547484

[B3] Au K. F. Underwood J. G. Lee L. Wong W. H. (2012). Improving PacBio long read accuracy by short read alignment. *PLoS ONE* 7:e46679 10.1371/journal.pone.0046679 PMC3464235 23056399

[B4] Avramova V. AbdElgawad H. Zhang Z. Fotschki B. Casadevall R. Vergauwen L. (2015). Drought induces distinct growth response, protection, and recovery mechanisms in the maize leaf growth zone. *Plant Physiol.* 169 1382–1396. 10.1104/pp.15.00276 26297138 PMC4587441

[B5] Barbazuk W. B. (2010). A conserved alternative splicing event in plants reveals an ancient exonization of 5S rRNA that regulates TFIIIA. *RNA Biol.* 7 397–402. 10.4161/rna.7.4.12684 20699638 PMC3070908

[B6] Barbazuk W. B. Fu Y. McGinnis K. M. (2008). Genome-wide analyses of alternative splicing in plants: opportunities and challenges. *Genome Res.* 18 1381–1392. 10.1101/gr.053678.106 18669480

[B7] Barbazuk W. B. Mei W. (2014). Genome sequencing. *Compend. Bioenergy Plants* 227–254.

[B8] Benjamini Y. Hochberg Y. (1995). Controlling the false discovery rate: a practical and powerful approach to multiple testing. *J. R. Stat. Soc. Ser. B* 57 289–300.

[B9] Bolger A. M. Lohse M. Usadel B. (2014). Trimmomatic: a flexible trimmer for Illumina sequence data. *Bioinformatics* 30 2114–2120. 10.1093/bioinformatics/btu170 24695404 PMC4103590

[B10] Braunschweig U. Barbosa-Morais N. L. Pan Q. Nachman E. N. Alipanahi B. Gonatopoulos-Pournatzis T. (2014). Widespread intron retention in mammals functionally tunes transcriptomes. *Genome Res.* 24 1774–1786. 10.1101/gr.177790.114 25258385 PMC4216919

[B11] Braunschweig U. Gueroussov S. Plocik A. M. Graveley B. R. Blencowe B. J. (2013). Dynamic integration of splicing within gene regulatory pathways. *Cell* 152 1252–1269. 10.1016/j.cell.2013.02.034 23498935 PMC3642998

[B12] Brown J. W. Simpson C. G. Marquez Y. Gadd G. M. Barta A. Kalyna M. (2015). Lost in translation: pitfalls in deciphering plant alternative splicing transcripts. *Plant Cell* 27 2083–2087. 10.1105/tpc.15.00572 26286536 PMC4568512

[B13] Busch A. Hertel K. J. (2012). Evolution of SR protein and hnRNP splicing regulatory factors. *Wiley Interdiscip. Rev. RNA* 3 1–12. 10.1002/wrna.100 21898828 PMC3235224

[B14] Capovilla G. Pajoro A. Immink R. G. Schmid M. (2015). Role of alternative pre-mRNA splicing in temperature signaling. *Curr. Opin. Plant Biol.* 27 97–103. 10.1016/j.pbi.2015.06.016 26190743

[B15] Chamala S. Feng G. Chavarro C. Barbazuk W. B. (2015). Genome-wide identification of evolutionarily conserved alternative splicing events in flowering plants. *Front. Bioeng. Biotechnol.* 3:33 10.3389/fbioe.2015.00033 PMC4374538 25859541

[B16] Chang C.-Y. Lin W.-D. Tu S.-L. (2014). Genome-wide analysis of heat-sensitive alternative splicing in *Physcomitrella patens*. *Plant Physiol.* 165 826–840. 10.1104/pp.113.230540 24777346 PMC4044832

[B17] Chen J. Zeng B. Zhang M. Xie S. Wang G. Hauck A. (2014). Dynamic transcriptome landscape of maize embryo and endosperm development. *Plant Physiol.* 166 252–264. 10.1104/pp.114.240689 25037214 PMC4149711

[B18] Drechsel G. Kahles A. Kesarwani A. K. Stauffer E. Behr J. Drewe P. (2013). Nonsense-mediated decay of alternative precursor mRNA splicing variants is a major determinant of the *Arabidopsis* steady state transcriptome. *Plant Cell* 25 3726–3742. 10.1105/tpc.113.115485 24163313 PMC3877825

[B19] Du Z. Zhou X. Ling Y. Zhang Z. Su Z. (2010). agriGO: a GO analysis toolkit for the agricultural community. *Nucleic Acids Res.* 38 W64–W70. 10.1093/nar/gkq310 20435677 PMC2896167

[B20] Dugas D. V. Monaco M. K. Olson A. Klein R. R. Kumari S. Ware D. (2011). Functional annotation of the transcriptome of *Sorghum bicolor* in response to osmotic stress and abscisic acid. *BMC Genomics* 12:1 10.1186/1471-2164-12-514 PMC3219791 22008187

[B21] Duvick J. Fu A. Muppirala U. Sabharwal M. Wilkerson M. D. Lawrence C. J. (2008). PlantGDB: a resource for comparative plant genomics. *Nucleic Acids Res.* 36 D959–D965. 10.1093/nar/gkm1041 18063570 PMC2238959

[B22] Eddy S. R. (1998). Profile hidden Markov models. *Bioinformatics* 14 755–763. 10.1093/bioinformatics/14.9.755 9918945

[B23] Filichkin S. A. Priest H. D. Givan S. A. Shen R. K. Bryant D. W. Fox S. E. (2010). Genome-wide mapping of alternative splicing in *Arabidopsis thaliana*. *Genome Res.* 20 45–58. 10.1101/gr.093302.109 19858364 PMC2798830

[B24] Florea L. Song L. Salzberg S. L. (2013). Thousands of exon skipping events differentiate among splicing patterns in sixteen human tissues. *F1000Res.* 2:188 10.12688/f1000research.2-188.v2 PMC3892928 24555089

[B25] Fouquet R. Martin F. Fajardo D. S. Gault C. M. Gómez E. Tseung C.-W. (2011). Maize rough endosperm3 encodes an RNA splicing factor required for endosperm cell differentiation and has a nonautonomous effect on embryo development. *Plant Cell* 23 4280–4297. 10.1105/tpc.111.092163 22138152 PMC3269866

[B26] Fu X.-D. Ares M. Jr. (2014). Context-dependent control of alternative splicing by RNA-binding proteins. *Nat. Rev. Genet.* 15 689–701. 10.1038/nrg3778 25112293 PMC4440546

[B27] Fu Y. Bannach O. Chen H. Teune J.-H. Schmitz A. Steger G. (2009). Alternative splicing of anciently exonized 5S rRNA regulates plant transcription factor TFIIIA. *Genome Res.* 19 913–921. 10.1101/gr.086876.108 19211543 PMC2675980

[B28] Gault C. M. Martin F. Mei W. Bai F. Black J. B. Barbazuk W. B. (2017). Aberrant splicing in maize *rough endosperm3* reveals a conserved role for U12 splicing in eukaryotic multicellular development. *Proc. Natl. Acad. Sci. U.S.A.* 114 E2195–E2204. 10.1073/pnas.1616173114 28242684 PMC5358371

[B29] Grabherr M. G. Haas B. J. Yassour M. Levin J. Z. Thompson D. A. Amit I. (2011). Full-length transcriptome assembly from RNA-Seq data without a reference genome. *Nat. Biotechnol.* 29 644–652. 10.1038/nbt.1883 21572440 PMC3571712

[B30] Graveley B. R. (2001). Alternative splicing: increasing diversity in the proteomic world. *Trends Genet.* 17 100–107. 10.1016/S0168-9525(00)02176-4 11173120

[B31] Haas B. J. Delcher A. L. Mount S. M. Wortman J. R. Smith R. K. Jr. Hannick L. I. (2003). Improving the *Arabidopsis* genome annotation using maximal transcript alignment assemblies. *Nucleic Acids Res.* 31 5654–5666. 10.1093/nar/gkg770 14500829 PMC206470

[B32] Hirsch C. N. Foerster J. M. Johnson J. M. Sekhon R. S. Muttoni G. Vaillancourt B. (2014). Insights into the maize pan-genome and pan-transcriptome. *Plant Cell* 26 121–135. 10.1105/tpc.113.119982 24488960 PMC3963563

[B33] Hughes T. E. Langdale J. A. Kelly S. (2014). The impact of widespread regulatory neofunctionalization on homeolog gene evolution following whole-genome duplication in maize. *Genome Res.* 24 1348–1355. 10.1101/gr.172684.114 24788921 PMC4120087

[B34] James A. B. Syed N. H. Bordage S. Marshall J. Nimmo G. A. Jenkins G. I. (2012). Alternative splicing mediates responses of the *Arabidopsis* circadian clock to temperature changes. *Plant Cell* 24 961–981. 10.1105/tpc.111.093948 22408072 PMC3336117

[B35] Kakumanu A. Ambavaram M. M. Klumas C. Krishnan A. Batlang U. Myers E. (2012). Effects of drought on gene expression in maize reproductive and leaf meristem tissue revealed by RNA-Seq. *Plant Physiol.* 160 846–867. 10.1104/pp.112.200444 22837360 PMC3461560

[B36] Kalyna M. Simpson C. G. Syed N. H. Lewandowska D. Marquez Y. Kusenda B. (2012). Alternative splicing and nonsense-mediated decay modulate expression of important regulatory genes in *Arabidopsis*. *Nucleic Acids Res.* 40 2454–2469. 10.1093/nar/gkr932 22127866 PMC3315328

[B37] Kazan K. (2003). Alternative splicing and proteome diversity in plants: the tip of the iceberg has just emerged. *Trends Plant Sci.* 8 468–471. 10.1016/j.tplants.2003.09.001 14557042

[B38] Kesari R. Lasky J. R. Villamor J. G. Des Marais D. L. Chen Y.-J. C. Liu T.-W. (2012). Intron-mediated alternative splicing of *Arabidopsis P5CS1* and its association with natural variation in proline and climate adaptation. *Proc. Natl. Acad. Sci. U.S.A.* 109 9197–9202. 10.1073/pnas.1203433109 22615385 PMC3384178

[B39] Lewis B. P. Green R. E. Brenner S. E. (2003). Evidence for the widespread coupling of alternative splicing and nonsense-mediated mRNA decay in humans. *Proc. Natl. Acad. Sci. U.S.A.* 100 189–192. 10.1073/pnas.0136770100 12502788 PMC140922

[B40] Li L. Petsch K. Shimizu R. Liu S. Xu W. W. Ying K. (2013). Mendelian and non-Mendelian regulation of gene expression in maize. *PLoS Genet.* 9:e1003202 10.1371/journal.pgen.1003202 PMC3547793 23341782

[B41] Liu S. Ying K. Yeh C. T. Yang J. Swanson-Wagner R. Wu W. (2012). Changes in genome content generated via segregation of non-allelic homologs. *Plant J.* 72 390–399. 10.1111/j.1365-313X.2012.05087.x 22731681

[B42] Makarevitch I. Waters A. J. West P. T. Stitzer M. Hirsch C. N. Ross-Ibarra J. (2015). Transposable elements contribute to activation of maize genes in response to abiotic stress. *PLoS Genet.* 11:e1004915 10.1371/journal.pgen.1004915 PMC4287451 25569788

[B43] Marquez Y. Brown J. W. S. Simpson C. Barta A. Kalyna M. (2012). Transcriptome survey reveals increased complexity of the alternative splicing landscape in *Arabidopsis*. *Genome Res.* 22 1184–1195. 10.1101/gr.134106.111 22391557 PMC3371709

[B44] Martin J. A. Johnson N. V. Gross S. M. Schnable J. Meng X. D. Wang M. (2014). A near complete snapshot of the *Zea mays* seedling transcriptome revealed from ultra-deep sequencing. *Sci. Rep.* 4:4519 10.1038/srep04519 PMC3970191 24682209

[B45] Martin M. (2011). Cutadapt removes adapter sequences from high-throughput sequencing reads. *EMBnet J.* 17 10–12. 10.14806/ej.17.1.200

[B46] Merkin J. Russell C. Chen P. Burge C. B. (2012). Evolutionary dynamics of gene and isoform regulation in mammalian tissues. *Science* 338 1593–1599. 10.1126/science.1228186 23258891 PMC3568499

[B47] Nagy E. Maquat L. E. (1998). A rule for termination-codon position within intron-containing genes: when nonsense affects RNA abundance. *Trends Biochem. Sci.* 23 198–199. 10.1016/S0968-0004(98)01208-0 9644970

[B48] Ni T. Yang W. Han M. Zhang Y. Shen T. Nie H. (2016). Global intron retention mediated gene regulation during CD4+ T cell activation. *Nucleic Acids Res.* 44 6817–6829. 10.1093/nar/gkw591 27369383 PMC5001615

[B49] Olson A. Klein R. R. Dugas D. V. Lu Z. Regulski M. Klein P. E. (2014). Expanding and vetting gene annotations through transcriptome and methylome Sequencing. *Plant Genome* 7 10.3835/plantgenome2013.08.0025

[B50] Opitz N. Paschold A. Marcon C. Malik W. A. Lanz C. Piepho H.-P. (2014). Transcriptomic complexity in young maize primary roots in response to low water potentials. *BMC Genomics* 15:1 10.1186/1471-2164-15-741 PMC4174653 25174417

[B51] Pan Q. Shai O. Lee L. J. Frey B. J. Blencowe B. J. (2008). Deep surveying of alternative splicing complexity in the human transcriptome by high-throughput sequencing. *Nat. Genet.* 40 1413–1415. 10.1038/ng.259 18978789

[B52] Paschold A. Jia Y. Marcon C. Lund S. Larson N. B. Yeh C. T. (2012). Complementation contributes to transcriptome complexity in maize (*Zea mays* L.) hybrids relative to their inbred parents. *Genome Res.* 22 2445–2454. 10.1101/gr.138461.112 23086286 PMC3514674

[B53] Paschold A. Larson N. B. Marcon C. Schnable J. C. Yeh C.-T. Lanz C. (2014). Nonsyntenic genes drive highly dynamic complementation of gene expression in maize hybrids. *Plant Cell* 26 3939–3948. 10.1105/tpc.114.130948 25315323 PMC4247586

[B54] Pertea M. Pertea G. M. Antonescu C. M. Chang T.-C. Mendell J. T. Salzberg S. L. (2015). StringTie enables improved reconstruction of a transcriptome from RNA-seq reads. *Nat. Biotechnol.* 33 290–295. 10.1038/nbt.3122 25690850 PMC4643835

[B55] Pimentel H. Parra M. Gee S. L. Mohandas N. Pachter L. Conboy J. G. (2016). A dynamic intron retention program enriched in RNA processing genes regulates gene expression during terminal erythropoiesis. *Nucleic Acids Res.* 44 838–851. 10.1093/nar/gkv1168 26531823 PMC4737145

[B56] Pophaly S. D. Tellier A. (2015). Population level purifying selection and gene expression shape subgenome evolution in maize. *Mol. Biol. Evol.* 32 3226–3235. 10.1093/molbev/msv191 26374232

[B57] Posé D. Verhage L. Ott F. Yant L. Mathieu J. Angenent G. C. (2013). Temperature-dependent regulation of flowering by antagonistic FLM variants. *Nature* 503 414–417. 10.1038/nature12633 24067612

[B58] Procissi A. Piazza P. Tonelli C. (2002). A maize r1 gene is regulated post-transcriptionally by differential splicing of its leader. *Plant Mol. Biol.* 49 239–248. 10.1023/A:1014959230492 11999378

[B59] Quinlan A. R. Hall I. M. (2010). BEDTools: a flexible suite of utilities for comparing genomic features. *Bioinformatics* 26 841–842. 10.1093/bioinformatics/btq033 20110278 PMC2832824

[B60] Rauch H. B. Patrick T. L. Klusman K. M. Battistuzzi F. U. Mei W. Brendel V. P. (2014). Discovery and expression analysis of alternative splicing events conserved among plant SR proteins. *Mol. Biol. Evol.* 31 605–613. 10.1093/molbev/mst238 24356560

[B61] Reddy A. S. N. Rogers M. F. Richardson D. N. Hamilton M. Ben-Hur A. (2012). Deciphering the plant splicing code: experimental and computational approaches for predicting alternative splicing and splicing regulatory elements. *Front. Plant Sci.* 3:18 10.3389/fpls.2012.00018 PMC3355732 22645572

[B62] Renaudin J.-P. Colasanti J. Rime H. Yuan Z. Sundaresan V. (1994). Cloning of four cyclins from maize indicates that higher plants have three structurally distinct groups of mitotic cyclins. *Proc. Natl. Acad. Sci. U.S.A.* 91 7375–7379. 10.1073/pnas.91.15.7375 8041798 PMC44402

[B63] Sabelli P. A. Larkins B. A. (2009). The development of endosperm in grasses. *Plant Physiol.* 149 14–26. 10.1104/pp.108.129437 19126691 PMC2613697

[B64] Satyawan D. Kim M. Y. Lee S. H. (2016). Stochastic alternative splicing is prevalent in mungbean (*Vigna radiata*). *Plant Biotechnol. J.* 15 174–182. 10.1111/pbi.12600 27400146 PMC5258860

[B65] Schnable J. C. Freeling M. (2011). Genes identified by visible mutant phenotypes show increased bias toward one of two subgenomes of maize. *PLoS ONE* 6:e17855 10.1371/journal.pone.0017855 PMC3053395 21423772

[B66] Schnable J. C. Springer N. M. Freeling M. (2011). Differentiation of the maize subgenomes by genome dominance and both ancient and ongoing gene loss. *Proc. Natl. Acad. Sci. U.S.A.* 108 4069–4074. 10.1073/pnas.1101368108 21368132 PMC3053962

[B67] Schnable P. S. Springer N. M. (2013). Progress toward understanding heterosis in crop plants. *Annu. Rev. Plant Biol.* 64 71–88. 10.1146/annurev-arplant-042110-103827 23394499

[B68] Schnable P. S. Ware D. Fulton R. S. Stein J. C. Wei F. Pasternak S. (2009). The B73 maize genome: complexity, diversity, and dynamics. *Science* 326 1112–1115. 10.1126/science.1178534 19965430

[B69] Sharon D. Tilgner H. Grubert F. Snyder M. (2013). A single-molecule long-read survey of the human transcriptome. *Nat. Biotechnol.* 31 1009–1014. 10.1038/nbt.2705 24108091 PMC4075632

[B70] Shen Y. Zhou Z. Wang Z. Li W. Fang C. Wu M. (2014). Global dissection of alternative splicing in paleopolyploid soybean. *Plant Cell* 26 996–1008. 10.1105/tpc.114.122739 24681622 PMC4001406

[B71] Shimada S. Makita Y. Kuriyama-Kondou T. Kawashima M. Mochizuki Y. Hirakawa H. (2015). Functional and expression analyses of transcripts based on full-length cDNAs of *Sorghum bicolor*. *DNA Res.* 22 485–493. 10.1093/dnares/dsv030 26546227 PMC4675717

[B72] Springer N. M. Ying K. Fu Y. Ji T. Yeh C.-T. Jia Y. (2009). Maize inbreds exhibit high levels of copy number variation (CNV) and presence/absence variation (PAV) in genome content. *PLoS Genet.* 5:e1000734 10.1371/journal.pgen.1000734 PMC2780416 19956538

[B73] Staiger D. Brown J. W. (2013). Alternative splicing at the intersection of biological timing, development, and stress responses. *Plant Cell* 25 3640–3656. 10.1105/tpc.113.113803 24179132 PMC3877812

[B74] Steijger T. Abril J. F. Engström P. G. Kokocinski F. Hubbard T. J. Guigó R. (2013). Assessment of transcript reconstruction methods for RNA-seq. *Nat. Methods* 10 1177–1184. 10.1038/nmeth.2714 24185837 PMC3851240

[B75] Stelpflug S. C. Sekhon R. S. Vaillancourt B. Hirsch C. N. Buell C. R. de Leon N. (2015). An expanded maize gene expression atlas based on RNA sequencing and its use to explore root development. *Plant Genome* 9 10.3835/plantgenome2015.04.0025 27898762

[B76] Streitner C. Simpson C. G. Shaw P. Danisman S. Brown J. W. Staiger D. (2013). Small changes in ambient temperature affect alternative splicing in *Arabidopsis thaliana*. *Plant Signal. Behav.* 8 11240–11255. 10.4161/psb.24638 PMC3907436 23656882

[B77] Sturgill D. Malone J. H. Sun X. Smith H. E. Rabinow L. Samson M.-L. (2013). Design of RNA splicing analysis null models for post hoc filtering of *Drosophila* head RNA-Seq data with the splicing analysis kit (Spanki). *BMC Bioinformatics* 14:320 10.1186/1471-2105-14-320 PMC3827500 24209455

[B78] Sugliani M. Brambilla V. Clerkx E. J. Koornneef M. Soppe W. J. (2010). The conserved splicing factor SUA controls alternative splicing of the developmental regulator *ABI3* in *Arabidopsis*. *Plant Cell* 22 1936–1946. 10.1105/tpc.110.074674 20525852 PMC2910958

[B79] Swanson-Wagner R. A. Eichten S. R. Kumari S. Tiffin P. Stein J. C. Ware D. (2010). Pervasive gene content variation and copy number variation in maize and its undomesticated progenitor. *Genome Res.* 20 1689–1699. 10.1101/gr.109165.110 21036921 PMC2989995

[B80] Swigonova Z. Lai J. S. Ma J. X. Ramakrishna W. Llaca V. Bennetzen J. L. (2004). Close split of sorghum and maize genome progenitors. *Genome Res.* 14 1916–1923. 10.1101/gr.2332504 15466289 PMC524415

[B81] Szécsi J. Joly C. Bordji K. Varaud E. Cock J. M. Dumas C. (2006). *BIGPETALp*, a *bHLH* transcription factor is involved in the control of *Arabidopsis* petal size. *EMBO J.* 25 3912–3920. 10.1038/sj.emboj.7601270 16902407 PMC1553195

[B82] Thatcher S. R. Danilevskaya O. N. Meng X. Beatty M. Zastrow-Hayes G. Harris C. (2016). Genome-wide analysis of alternative splicing during development and drought stress in maize. *Plant Physiol.* 170 586–599. 10.1104/pp.15.01267 26582726 PMC4704579

[B83] Thatcher S. R. Zhou W. Leonard A. Wang B.-B. Beatty M. Zastrow-Hayes G. (2014). Genome-wide analysis of alternative splicing in *Zea mays*: landscape and genetic regulation. *Plant Cell* 26 3472–3487. 10.1105/tpc.114.130773 25248552 PMC4213170

[B84] Trapnell C. Pachter L. Salzberg S. L. (2009). TopHat: discovering splice junctions with RNA-Seq. *Bioinformatics* 25 1105–1111. 10.1093/bioinformatics/btp120 19289445 PMC2672628

[B85] Trapnell C. Roberts A. Goff L. Pertea G. Kim D. Kelley D. R. (2012). Differential gene and transcript expression analysis of RNA-seq experiments with TopHat and Cufflinks. *Nat. Protoc.* 7 562–578. 10.1038/nprot.2012.016 22383036 PMC3334321

[B86] Varagona M. J. Purugganan M. Wessler S. R. (1992). Alternative splicing induced by insertion of retrotransposons into the maize waxy gene. *Plant Cell* 4 811–820. 10.1105/tpc.4.7.811 1327340 PMC160176

[B87] Wang B. Tseng E. Regulski M. Clark T. A. Hon T. Jiao Y. (2016). Unveiling the complexity of the maize transcriptome by single-molecule long-read sequencing. *Nat. Commun.* 7:11708 10.1038/ncomms11708 PMC4931018 27339440

[B88] Wang E. T. Sandberg R. Luo S. Khrebtukova I. Zhang L. Mayr C. (2008). Alternative isoform regulation in human tissue transcriptomes. *Nature* 456 470–476. 10.1038/nature07509 18978772 PMC2593745

[B89] Wang Z. Burge C. B. (2008). Splicing regulation: from a parts list of regulatory elements to an integrated splicing code. *RNA* 14 802–813. 10.1261/rna.876308 18369186 PMC2327353

[B90] Ward J. H. Jr. (1963). Hierarchical grouping to optimize an objective function. *J. Am. Stat. Assoc.* 58 236–244. 10.1080/01621459.1963.10500845

[B91] Wu T. D. Nacu S. (2010). Fast and SNP-tolerant detection of complex variants and splicing in short reads. *Bioinformatics* 26 873–881. 10.1093/bioinformatics/btq057 20147302 PMC2844994

[B92] Wu T. D. Watanabe C. K. (2005). GMAP: a genomic mapping and alignment program for mRNA and EST sequences. *Bioinformatics* 21 1859–1875. 10.1093/bioinformatics/bti310 15728110

[B93] Xiong H. Y. Alipanahi B. Lee L. J. Bretschneider H. Merico D. Yuen R. K. (2015). The human splicing code reveals new insights into the genetic determinants of disease. *Science* 347:1254806 10.1126/science.1254806 PMC4362528 25525159

[B94] Yeo G. W. Nostrand E. Liang T. Y. (2007). Discovery and analysis of evolutionarily conserved intronic splicing regulatory elements. *PLoS Genet.* 3:e85 10.1371/journal.pgen.0030085 PMC1877881 17530930

[B95] Yilmaz A. Nishiyama M. Y. Jr. Fuentes B. G. Souza G. M. Janies D. Gray J. (2009). GRASSIUS: a platform for comparative regulatory genomics across the grasses. *Plant Physiol.* 149 171–180. 10.1104/pp.108.128579 18987217 PMC2613736

[B96] Zhang X. Cal A. J. Borevitz J. O. (2011). Genetic architecture of regulatory variation in *Arabidopsis thaliana*. *Genome Res.* 21 725–733. 10.1101/gr.115337.110 21467266 PMC3083089

