# Supplementary material for: A Comprehensive Analysis of Alternative Splicing in Paleopolyploid Maize
Source: Front Plant Sci. 2017 May 10;8:694. doi: 10.3389/fpls.2017.00694 (PMC5423905; doi:10.3389/fpls.2017.00694)
Supplement: Supplementary file 1 [file Data_Sheet_1.DOCX]

Supplementary Material

**A Comprehensive Analysis of Alternative Splicing in Paleopolyploid Maize**

**Wenbin Mei^1^, Sanzhen Liu^2, 3^, James C. Schnable^4^, Cheng-Ting Yeh^2^, Nathan M. Springer^5^, Patrick S. Schnable^2, 7^, W. Brad Barbazuk^1, 6,^ ***

^1^Department of Biology, University of Florida, Gainesville, Florida, USA.

^2^Department of Agronomy, Iowa State University, Ames, Iowa, USA.

^3^Department of Plant Pathology, Kansas State University, Manhattan, Kansas, USA.

^4^Agronomy & Horticulture, University of Nebraska–Lincoln, Beadle Center E207, Lincoln, Nebraska, USA.

^5^Department of Plant Biology, Microbial and Plant Genomics Institute, University of Minnesota, Saint Paul, Minnesota, USA.

^6^Genetics Institute, University of Florida, Gainesville, Florida, USA.

^7^Center for Plant Genomics, Iowa State University, Ames, Iowa, USA.

*** Correspondence:** W. Brad Barbazuk Department of Biology, University of Florida, Gainesville, USA Telephone: 1(352) 273-8624, Fax: 1(352) 273-8284, Email: bbarbazuk@ufl.edu

# Supplementary Data

**Supplementary Data 1**. Summary of RNA-Seq data used in B73, Mo17 and Sorghum.

**Supplementary Data 2**. Data Summary in each tissue in B73 and Mo17.

**Supplementary Data 3**. Alternative splicing in maize classic genes.

**Supplementary Data 4**. Alternative splicing pairwise comparision among 9 tissues.

**Supplementary Data 5**. Differential splicing of SR and hnRNP in response to heat and cold.

**Supplementary Data 6**. The list of genes show differential splicing in response to a variety of stress.

**Supplementary Data 7**. Present and absent isoforms between B73 vs. Mo17 in five tissues.

# Supplementary Figures and Tables

## Supplementary Figures

**Supplemental Figure 1. The pipeline to identify AS in inbred line B73.** The pipeline to generate maize B73 alternative splicing isoforms leverages three different assembly strategies and subsequent PASA clustering into potential full-length transcript isoforms. Additional steps used to filter out noise are listed.

**Supplemental Figure 2. Pearson correlation of IRR value (random subsampling vs. raw data) in different tissues.** A) Leaf tissue. B) Seed tissue. C) Silks tissue. D) Anther tissue.

**Supplemental Figure 3. Pearson correlation of PSI value (random subsampling vs. raw data) in different tissue.** A) Shoot tissue. B) Coleoptile tissue. C) Ear tissue. D) Root tissue.

**Supplemental Figure 4. Characterization of AS isoforms.** A) The proportion of AS events (expressed as %) distributed within the 5’ UTR, gene body or 3’ UTR respectively. B) The proportion of AS isoforms (expressed as %) predicted to result in a reading frame shift relative to the non-AS transcript. C) The proportion of AS isoforms (expressed as %) predicted to result in a domain change relative to the non-AS transcript. D) The proportion of AS isoforms (expressed as %) predicted to be potential candidates of nonsense-mediated decay based on criteria (Nagy and Maquat, 1998).

**Supplemental Figure 5. AS in transcription factor families.** Percentage of genes that undergo AS vs. number of AS genes in transcription factor families, the points annotated in red represent transcription factor families with more alternative splicing. The histogram on the top represents the distribution of number of AS genes and the histogram on the right represents the distribution of percentage of genes undergo AS.

**Supplemental Figure 6. Number of AS genes and percentage of AS events in B73 18 tissues.** A) Number of genes that exhibit AS on a tissue-by-tissue basis. B) Percentage of AS event on a tissue-by-tissue basis. All counted AS events require skip exon and intron retention isoforms minimal FPKM of 1 and for the rest of AS events, both isoforms needs to above 1.

**Supplemental Figure 7. Significant GO term enrichment associated with genes producing intron retention isoforms across seed development with ΔIRR (38 day – 0 day) > 0.15.** GO term analysis was performed with agriGO (Methods) using Singular Enrichment Analysis by Fisher test with Hochberg (FDR) control at minimal q-value of 0.05.

**Supplemental Figure 8. GO term enrichment of genes differentially spliced in response to both heat (vs. control) and cold (vs. control) stress.** GO term analysis was performed with agriGO (Methods) using a Singular Enrichment Analysis by Fisher test, with a Hochberg (FDR) control of minimal q value of 0.05.

**Supplemental Figure 9. Integrative Genomics Viewer (IGV) of maize locus zm-SC30 (GRMZM2G016296) in five different stresses.** Intron Retention identified is identified within the 3’ UTR region, and the location the intron retention event is indicated with red arrows. Two isoforms (GRMZM2G016296_asmbl_188362 and GRMZM2G016296_asmbl_188364) spliced out the 3’ UTR intron, but would lead to nonsense mediate decay.

## Supplementary Table

**Supplemental Table 1. Five alternative splicing types from maize and sorghum calculated in genes, isoforms and events.** The percentage is calculated based on each AS type counts divided by the total number respectively. alternative acceptor: AltA; alternative donor: AltD; Exon Skip: ExonS; Intron Retention: IntronR; alternate terminal exon: AltTE.

| **AS type** |  | **B73** | **Mo17** | **Sorghum** |
| --- | --- | --- | --- | --- |
| AltA | Genes (%)  Isoforms (%)  Events (%) | 6,006 (19.5)  21,028 (42.0)  9,890 (18.4) | 4,902 (15.3)  17,840 (45.5)  8,200 (20.7) | 2,959 (12.4)  8,587 (44.2)  4,248 (27.0) |
| AltD | Genes (%)  Isoforms (%)  Events (%) | 4,743 (15.4)  16,624 (33.2)  7,557 (14.1) | 3,746 (11.7)  13,612 (34.7)  5,969 (15.1) | 2,000 (8.4)  5,898 (30.4)  2,730 (17.3) |
| AltTE | Genes (%)  Isoforms (%)  Events (%) | 1,818 (5.9)  5,976 (11.9)  2,744 (5.1) | 1,472 (4.6)  5,143 (13.1)  2,207 (5.6) | 1,270 (5.3)  3,601 (18.6)  1,707 (10.8) |
| ExonS | Genes (%)  Isoforms (%)  Events (%) | 2,779 (9.0)  5,543 (11.1)  3,619 (6.7) | 2,424 (7.6)  4,821 (12.3)  3,101 (7.8) | 1,207 (5.1)  1,936 (10.0)  1,457 (9.3) |
| IntronR | Genes (%)  Isoforms (%)  Events (%) | 10,423 (33.9)  23,588 (47.1)  29,882 (55.7) | 7,620 (23.8)  16,573 (42.2)  20,148 (50.8) | 3,030 (12.7)  5,113 (26.3)  5,602 (35.6) |
| Total | Genes  Isoforms  Events | 14,321 (46.6)  50,083  53,692 | 11,299 (35.3)  39,248  39,625 | 6,839 (28.7)  19,407  15,744 |

**Supplemental Table 2. AS number and frequency in splicing related genes.**

|  | Genes | AltA | AltD | AltTE | ExonS | IntronR | Overall | Percentage |
| --- | --- | --- | --- | --- | --- | --- | --- | --- |
| snRNP_proteins | 76 | 24 | 13 | 8 | 8 | 51 | 38 | 50.0% |
| splicing_factor | 103 | 64 | 73 | 20 | 70 | 716 | 62 | 60.2% |
| splicing_regulation | 52 | 28 | 21 | 4 | 16 | 106 | 42 | 80.8% |

**Supplemental Table 3.** Expression levels for isoforms from *cyc3* (GRMZM2G073671) under drought stress. The expression level (FPKM) is calculated in Cufflinks. Intron spiced isoform GRMZM2G073671_asmbl_272716 is marked with an asterisk (*). We calculate the percentage of expression levels of GRMZM2G073671_asmbl_272716 relative to gene expression level.

|  | 6h mild | 6h severe | 24h mild | 24h severe | 6h control | 24h control |
| --- | --- | --- | --- | --- | --- | --- |
| GRMZM2G073671_asmbl_272715 | 9.82 | 10.46 | 11.61 | 11.77 | 8.95 | 9.90 |
| GRMZM2G073671_asmbl_272716* | 0.15 | 0.00 | 0.49 | 0.00 | 3.48 | 4.88 |
| GRMZM2G073671_asmbl_272717 | 0.16 | 0.00 | 0.00 | 0.00 | 0.00 | 0.44 |
| GRMZM2G073671_asmbl_272718 | 0.81 | 0.62 | 0.69 | 0.39 | 0.71 | 1.14 |
| GRMZM2G073671 | 10.94 | 11.08 | 12.79 | 12.15 | 13.13 | 16.36 |
| asmbl_272716/gene expression % | 0.01 | 0.00 | 0.04 | 0.00 | 0.26 | 0.30 |

**Supplemental Table 4.** Expression levels for isoforms from zm-SC30 (GRMZM2G016296) in five stresses condition. The expression level (FPKM) is calculated in Cufflinks. GRMZM2G016296_asmbl_188362 and GRMZM2G016296_asmbl_188364 (spliced intron isoform) generated one additional intron at 3’ UTR region and would lead to nonsense mediate decay marked with an asterisk (*). We calculate the percentage of GRMZM2G016296_asmbl_188362 plus GRMZM2G016296_asmbl_188364 relative to gene expression level.

|  | Control | Cold | Heat | Salt | UV |
| --- | --- | --- | --- | --- | --- |
| GRMZM2G016296_asmbl_188360 | 1.07 | 0.55 | 0.77 | 1.77 | 1.86 |
| GRMZM2G016296_asmbl_188361 | 6.53 | 9.49 | 14.33 | 21.77 | 0.01 |
| GRMZM2G016296_asmbl_188362* | 5.31 | 2.72 | 1.55 | 3.31 | 2.75 |
| GRMZM2G016296_asmbl_188363 | 7.88 | 4.23 | 1.97 | 2.53 | 1.32 |
| GRMZM2G016296_asmbl_188364* | 26.77 | 16.03 | 16.02 | 8.51 | 9.20 |
| GRMZM2G016296_asmbl_188365 | 0.28 | 0.66 | 1.28 | 0.00 | 12.42 |
| GRMZM2G016296_asmbl_188366 | 4.74 | 7.93 | 6.65 | 11.88 | 4.52 |
| GRMZM2G016296_asmbl_188367 | 0.15 | 0.42 | 0.19 | 0.00 | 0.00 |
| GRMZM2G016296 | 52.73 | 42.03 | 42.76 | 49.77 | 32.08 |
| (asmbl_188362+asmbl_188364)/gene expression % | 60.8% | 44.6% | 41.1% | 23.7% | 37.2% |
