# Supplementary material for: A Comprehensive Analysis of Alternative Splicing in Paleopolyploid Maize
Source: Front Plant Sci. 2017 May 10;8:694. doi: 10.3389/fpls.2017.00694 (PMC5423905; doi:10.3389/fpls.2017.00694)
Supplement: Supplementary file 3 [file Data_Sheet_2.DOCX]

Supplemental Data 1: The list of all RNA-Seq samples we used in this manuscript including 403 B73 samples, 66 Mo17 samples and 40 sorghum samples. Separate tables (pages) are included that detail the stress related data and seed development data. For each short reads SRA sample, we listed the SRA study name, read type, tissue type, the number of raw reads, the number of reads after Trimmomatic data cleaning steps, the number of reads aligned in the GSNAP software, the number of aligned read remaining after “remove duplicates” filtering.

Supplemental Data 2: The RNA-seq data summary for each tissue in B73 and Mo17, and the number of samples in each tissue. Raw read counts and the number of aligned read remaining after “remove duplicates” filtering for each tissue are presented.

Supplemental Data 3: The details of alternative splicing events and their location in the maize classic genes. Classical gene symbols, maize gene IDs and whether the locus resides within maize subgenome1 or subgenome2 are noted.

Supplemental Data 4: CUFFDIFF differential splicing metrics for loci with alternatively spliced isoforms from tissue vs. tissue comparisons. Each tales lists CUFFDIFF significance metrics, maize loci and tissue comparison. Pages are organized by tissue.

Supplemental Data 5: Differential splicing of maize SR protein and hnRNP in response to heat and cold stress. SR protein and hnRNP protein names and their maize locus IDs are listed.

Supplemental Data 6: The list of genes showed differential splicing in response to different stresses including ovary tissue (drought vs. control), leaf (drought vs. control), seedling (cold vs. control), seedling (heat vs. control), seedling primary root 6h (severe drought vs. control), seedling primary root 24h (mild drought vs. control), seedling primary root 24h (severe drought vs. control).

Supplemental Data 7: Present and absent isoforms between B73 vs. Mo17 comparison in leaf, root, shoot apical meristem, seed and seedling. The results are based on CUFFDIFF analysis.
